# Role of viscoelasticity in the appearance of low-Reynolds turbulence: considerations for modelling

**DOI:** 10.1186/s13036-024-00415-6

**Published:** 2024-04-08

**Authors:** Ivana Pajic-Lijakovic, Milan Milivojevic, Peter V. E. McClintock

**Affiliations:** 1 Faculty of Technology and Metallurgy, Department of Chemical Engineering, University of Belgrade, Belgrade, Serbia; 2 Department of Physics, Lancaster University, Lancaster, LA1 4YB UK

**Keywords:** Mechanical stress generation, Energy storage, Energy dissipation, Collective cell migration, Cell-jamming state transition, The strength of cell–cell and cell–matrix adhesion contacts

## Abstract

Inertial effects caused by perturbations of dynamical equilibrium during the flow of soft matter constitute a hallmark of turbulence. Such perturbations are attributable to an imbalance between energy storage and energy dissipation. During the flow of Newtonian fluids, kinetic energy can be both stored and dissipated, while the flow of viscoelastic soft matter systems, such as polymer fluids, induces the accumulation of both kinetic and elastic energies. The accumulation of elastic energy causes local stiffening of stretched polymer chains, which can destabilise the flow. Migrating multicellular systems are hugely complex and are capable of self-regulating their viscoelasticity and mechanical stress generation, as well as controlling their energy storage and energy dissipation. Since the flow perturbation of viscoelastic systems is caused by the inhomogeneous accumulation of elastic energy, rather than of kinetic energy, turbulence can occur at low Reynolds numbers.

This theoretical review is focused on clarifying the role of viscoelasticity in the appearance of low-Reynolds turbulence. Three types of system are considered and compared: (1) high-Reynolds turbulent flow of Newtonian fluids, (2) low and moderate-Reynolds flow of polymer solutions, and (3) migration of epithelial collectives, discussed in terms of two model systems. The models considered involve the fusion of two epithelial aggregates, and the free expansion of epithelial monolayers on a substrate matrix.

## Introduction

Turbulence is a common phenomenon in nature. The flows of air in the atmosphere, water in rivers and oceans, and a variety of fluids in industrial plants, are just a few examples [1–3]. Physiological blood flow occurs under Reynolds numbers less than 2400 and consequently is usually considered as a laminar flow [4]. However, recent findings pointed out that the multi-harmonic nature of blood flow is the primary cause of turbulence [5]. Collective cell migration during morphogenesis has recently been recognised as a turbulent phenomenon as well [6–11]. While turbulence in Newtonian fluids and in viscoelastic systems, such as polymer solutions, has been well elaborated [1–3, 12–14], turbulence in viscoelastic multicellular systems caused by collective cell migration is only starting to be elucidated. A deeper understanding of turbulence is necessary in order to predict and control natural processes, biomedical processes, tissue development and self-organisation, and a variety of processes in industry. Transition between laminar and turbulent flow occurs through various transient states. These transient states have not been properly characterized even for flow of Newtonian fluids [2]. Inertial effects, induced by fluid flow, represent a hallmark of turbulence. They are caused by the generation of flow instabilities in the form of eddies. The latter represent unstable supramolecular/supracellular structures which can be characterized by their kinetic energy, elastic energy, size, and lifetime. The formation of eddies depends on: (1) the energy input into the system (i.e. driving forces), (2) resistive forces, which are sometimes connected with energy dissipation, and (3) the system’s rheological behaviour. Consequently, inertial effects arise when the dynamic equilibrium between driving forces and resistive forces is perturbed. The driving force for the flow of Newtonian fluids is the pressure gradient, responsible for the input of kinetic energy, while the resistive force is represented by the divergence of the shear stress, responsible for the energy dissipation [3]. The turbulent flow of soft matter systems such as polymer solutions and multicellular systems depends in particular on the systems’ viscoelasticity [8, 15, 16]. While the flow of polymer solutions is induced by the pressure gradient, the flow of living matter such as movement of epithelial collectives can be driven by a range of extracellular signals. These include chemical, mechanical, electrical (influencing the cell contractility), and the strengths of cell–cell and cell–matrix adhesion contacts which together provide the basis for the viscoelasticity of multicellular systems [17, 18].

The turbulence which occurs under isothermal conditions, when gravity can be neglected, has been characterized by a range of dimensionless numbers including: (1) the Reynolds number (${R}_{e}$) – the ratio between the inertial and viscous forces, (2) the Weber number (${W}_{e}$) – the ratio between the kinetic and surface energies, and (3) the Weissenberg number (${W}_{i}$) which accounts for the viscoelasticity caused by shear flow [3]. The Reynolds number is defined as ${R}_{e}=\frac{{\rho }_{l}UL}{{\upeta }_{l}}$ (where ${\upeta }_{l}$ is the viscosity of fluid, ${\rho }_{l}$ is its density, $L$ is a characteristic length, $U=\frac{Q}{S}$ is the average velocity, $Q$ is the volumetric flow rate, and $S$ is the cross-sectional area). The Weissenberg number is defined as ${W}_{i}={\tau }_{RpV}\frac{U}{L}$ (where ${\tau }_{RpV}$ is the stress relaxation time for polymer chains). The Weber number for a fluid droplet is defined as ${W}_{e}=\frac{{\rho }_{l}{v}^{2}l}{{\gamma }_{l}}$ (where ${\gamma }_{l}$ is the fluid surface tension, $v$ is the droplet speed, and $l$ is the droplet diameter) [19]. This list is not final and should be extended to describe turbulence caused by the viscoelastic nature of the flowing systems considered [12].

The ${R}_{e}$ number has been used to characterize the flow of Newtonian fluids in various geometries. In many cases, such as the flow of fluid through a pipe, it is the only dimensionless number needed. When the flowing fluids have a free surface with air, however, such as a flow through open channels and fluid jets, it is also necessary to include the ${W}_{e}$ number. The ${R}_{e}$ number for turbulent flow of Newtonian fluids through pipes is ${R}_{e}>3500$. However, turbulent flow of Newtonian fluids through open channels can occur at lower ${R}_{e}$ values, i.e. ${R}_{e}>2000$. While fluid flow through a pipe consists of unperturbed core flow (without the velocity gradient) and flow within the boundary layers of fluid in contact with the wall (with the large velocity gradient), flow through open channels can be treated as the boundary layer only [3]. Energy dissipation caused by a fluid flow within boundary layers is more intensive than that due to flow through the core region of pipes. Consequently, the more dissipative flow of fluid through open channels is characterized by a lower ${R}_{e}$ number. Turbulence in the circular Couette flow of Newtonian fluids, for the case where only the inner cylinder rotates occurs for ${R}_{ei}>1500$ (where ${R}_{ei}$ is the Reynolds number at the inner cylinder) [2]. Fluid flow past a sphere induces flow separation, causing additional energy dissipation, and results in the generation of flow instabilities under an even smaller ${R}_{e}$ number, i.e. ${R}_{e}>10$ [3].

However, turbulent flow of viscoelastic systems such as polymer solutions consisting of long and flexible polymer chains occurs at low and moderate ${R}_{e}$ numbers [12, 15, 16]. In this context two types of turbulence have been discussed: elastic turbulence (i.e. the inertia-less turbulence obtained for ${R}_{e}<1$), and elasto-inertial turbulence (obtained in Couette flow for ${R}_{ei}\ge 200$), depending on the magnitude of the inertial force [20]. The elastic turbulence has been treated as some transient state [15]. In the case of elastic/elastic-inertial turbulence, an additional dimensionless number is introduced to characterise the system viscoelasticity. It is the Weissenberg number, ${W}_{i}$, which correlates the polymer stress relaxation time with the macroscopic shear rate. Collective cell migration is another example of low-${R}_{e}$ turbulence caused by the system’s viscoelasticity. However, in contrast to other soft matter systems, multicellular systems are active, and capable of self-rearranging, which has been treated as an active form of turbulence [11]. The viscoelasticity of multicellular systems caused by collective cell migration is a complex and multi-time phenomenon. Low-${R}_{e}$ turbulence, caused by movement of multicellular systems, has been recognised in model systems such as: (1) free expansion of epithelial monolayers [6, 8]; (2) the rearrangement of confluent epithelial monolayers [7]; (3) the fusion of two cell aggregates [21, 22]; (4) cell aggregate rounding after uni-axial compression between parallel plates [23, 24]; (5) cell aggregate wetting on rigid substrates [25]; and (6) the segregation of co-cultured epithelial-mesenchymal spheroids [26]. The ‘’active turbulence’’ is connected to long-time inertial effects (i.e. effective inertial effects) caused by: (1) inhomogeneous distribution of the cell-packing density, the cell velocity, the corresponding strain, and the cell residual stress; and (2) their oscillations on a time-scale of hours in the form of mechanical waves [6–8]. Consequently, the active turbulence is induced by density-driven changes in the state of viscoelasticity accompanied by tissue surface characteristics [6, 27, 28]. A detailed understanding of the turbulence, caused by collective cell migration, promises to facilitate the control of critical multicellular processes in development, regenerative medicine, and invasive diseases.

The main focus of the present review is: (1) to point to the role of viscoelasticity in the appearance of the low and moderate-${R}_{e}$ turbulence by considering the flow of polymer solutions and collective cell migration; and (2) to compare this type of turbulence with the high-${R}_{e}$ turbulence occurring during the flow of Newtonian fluids. A deeper insight into the main causes responsible for the appearance of active turbulence within the epithelium promises new ways to: (1) stimulate wound healing; (2) understand the orderly trend of tissue self-organisation required during morphogenesis; and (3) suppress the spreading of cancer through the epithelium.

## High-${{\varvec{R}}}_{{\varvec{e}}}$ turbulence in Newtonian fluids

Two types of turbulence will be considered and compared: (1) high-${R}_{e}$ turbulence and (2) low and moderate-${R}_{e}$ turbulence, respectively. As already mentioned, the onset of turbulence is connected with inertial effects. The underlying mechanisms responsible for generating the inertial effects are related mainly to the system’s rheological behaviour. While high-${R}_{e}$ turbulence occurs in Newtonian fluids, low and moderate-${R}_{e}$ turbulence occurs in viscoelastic systems such as polymer solutions made up of flexible, high molecular weight polymer chains, and in epithelial multicellular systems. First, the constitutive behaviour of Newtonian fluids will be described, and then the phenomenon will be discussed based on the equations for conservation of momentum and mass. For characterizing the turbulent flow of Newtonian fluids through pipes, only the ${R}_{e}$ number is needed. In order to appreciate the origin of the inertial effects, it is necessary to discuss the rheological behaviour of Newtonian fluids under turbulent flow and then to formulate the momentum and mass conservation equations.

### Newtonian fluids: their rheological behaviour

The shear flow of Newtonian fluids is purely dissipative. While the constitutive stress–strain model for laminar flow of Newtonian fluids is linear, the constitutive model for turbulent flow is non-linear. Its non-linearity arises from the fluctuations of fluid velocity. Moreover, the local velocity of turbulent flow $\overrightarrow{{{\varvec{v}}}_{{\varvec{l}}}}\left(r,t\right)={\overrightarrow{{\varvec{V}}}}_{{\varvec{l}}}+{\overrightarrow{{\varvec{v}}}}_{{\varvec{l}}}^{\boldsymbol{^{\prime}}}$ (where ${\overrightarrow{{\varvec{V}}}}_{{\varvec{l}}}$ is the average velocity and ${\overrightarrow{{\varvec{v}}}}_{{\varvec{l}}}^{\boldsymbol{^{\prime}}}$ is the fluctuating velocity that satisfies the condition that $\langle {\overrightarrow{{\varvec{v}}}}_{{\varvec{l}}}^{\boldsymbol{^{\prime}}}\rangle =0$). The generated stress includes two components, i.e.

1 $${\widetilde{{\varvec{\sigma}}}}_{{\varvec{l}}{\varvec{S}}}\left(r,t\right)={\upeta }_{l}{\dot{\widetilde{{\varvec{\varepsilon}}}}}_{{\varvec{l}}{\varvec{S}}}\left(r,t\right)+{{\widetilde{{\varvec{\sigma}}}}_{{\varvec{l}}{\varvec{S}}}}^{\boldsymbol{^{\prime}}}$$

where $r$ is the space coordinate, $t$ is time, ${\widetilde{{\varvec{\sigma}}}}_{{\varvec{l}}{\varvec{S}}}$ is the shear stress,${\dot{\widetilde{{\varvec{\varepsilon}}}}}_{{\varvec{l}}{\varvec{S}}}$ is the shear strain rate expressed for the average velocity in the form ${\dot{\widetilde{{\varvec{\varepsilon}}}}}_{{\varvec{l}}{\varvec{S}}}=\frac{1}{2}\left(\overrightarrow{\nabla }\overrightarrow{{{\varvec{V}}}_{{\varvec{l}}}}+{\overrightarrow{\nabla }\overrightarrow{{{\varvec{V}}}_{{\varvec{l}}}}}^{{\varvec{T}}}\right)$**,** ${\upeta }_{l}$ is the molecular viscosity and ${{\widetilde{{\varvec{\sigma}}}}_{{\varvec{l}}{\varvec{S}}}}^{\boldsymbol{^{\prime}}}$ is the Reynolds stress which arises as a consequence of the velocity fluctuations. The component of the Reynolds stress can be expressed as: ${{\widetilde{{\varvec{\sigma}}}}_{{\varvec{l}}{\varvec{S}}\boldsymbol{\varvec{i}}{\varvec{j}}}}^{\boldsymbol{^{\prime}}}={-\rho }_{l}\langle {\overrightarrow{{\varvec{v}}}}_{{\varvec{l}}{\varvec{i}}}^{\boldsymbol{^{\prime}}}{\overrightarrow{{\varvec{v}}}}_{{\varvec{l}}{\varvec{j}}}^{\boldsymbol{^{\prime}}}\rangle$** (**where ${\overrightarrow{{\varvec{v}}}}_{{\varvec{l}}{\varvec{i}}}^{\boldsymbol{^{\prime}}}$ and ${\overrightarrow{{\varvec{v}}}}_{{\varvec{l}}{\varvec{j}}}^{\boldsymbol{^{\prime}}}$ are components of fluctuating velocity and ${\rho }_{l}$ is the density of fluid) [1]. The first term on the right-hand side is the linear part of the stress, while the second is its non-linear part. The Reynolds stress can be described by introducing the eddy viscosity in the form: ${{\widetilde{{\varvec{\sigma}}}}_{{\varvec{l}}{\varvec{S}}}}^{\boldsymbol{^{\prime}}}={\upeta }_{e}\left(r,t\right){\dot{\widetilde{{\varvec{\varepsilon}}}}}_{{\varvec{l}}{\varvec{S}}}\left(r,t\right)$, where ${\upeta }_{e}\left(r,t\right)$ is the eddy viscosity. While the viscosity ${\upeta }_{l}$ of the Newtonian fluid remains constant under isothermal conditions, the viscosity ${\upeta }_{e}\left(r,t\right)$ is a hypothesised property of the flow and depends on the size and distribution of the eddies [3]. The limitations of the eddy-viscosity approach arise from its assumption of equilibrium between the turbulence and the mean strain field ${\dot{\widetilde{{\varvec{\varepsilon}}}}}_{{\varvec{l}}{\varvec{S}}}\left(r,t\right)$, and also from the assumed independence of system rotation [29]. The changes in stress and shear strain rate occur on the same time-scale. Newtonian fluids can adsorb kinetic energy even when gravity is neglected.

### Conservation of momentum and mass equations for the flow of Newtonian fluids

The simplest form of the conservation of momentum in the turbulent flow of incompressible, Newtonian fluid (i.e. $\overrightarrow{\nabla }\cdot {\overrightarrow{{\varvec{V}}}}_{{\varvec{l}}}=0$) through pipes can be expressed in the form of the Reynolds-averaged Navier–Stokes equation as [1]:

2 $${\rho }_{l}\frac{D{\overrightarrow{{\varvec{V}}}}_{{\varvec{l}}}}{Dt}={\overrightarrow{{\varvec{F}}}}_{{\varvec{d}}}-{\overrightarrow{{\varvec{F}}}}_{{\varvec{r}}}$$

where $\frac{D{\overrightarrow{{\varvec{V}}}}_{{\varvec{l}}}}{Dt}=\frac{\partial {\overrightarrow{{\varvec{V}}}}_{{\varvec{l}}}}{\partial t}+({\overrightarrow{{\varvec{V}}}}_{{\varvec{l}}}\cdot \overrightarrow{\nabla }){\overrightarrow{{\varvec{V}}}}_{{\varvec{l}}}$ is the material derivatives [1], the driving force for the fluid flow ${\overrightarrow{{\varvec{F}}}}_{{\varvec{d}}}=-\overrightarrow{\nabla }p$, $\overrightarrow{\nabla }p$ is the pressure gradient, and ${\overrightarrow{{\varvec{F}}}}_{{\varvec{r}}}$ is the resistive, dissipative force equal to ${\overrightarrow{{\varvec{F}}}}_{{\varvec{r}}}=\nabla \cdot {\widetilde{{\varvec{\sigma}}}}_{{\varvec{l}}{\varvec{S}}}$. The presence of eddies in the flow, quantified by the fluctuating part of the stress ${{\widetilde{{\varvec{\sigma}}}}_{{\varvec{l}}{\varvec{S}}}}^{\boldsymbol{^{\prime}}}$, causes continuous fluctuations of the dissipative, resistive force, leading to an imbalance between these two forces, i.e. ${\overrightarrow{{\varvec{F}}}}_{{\varvec{d}}}\ne {\overrightarrow{{\varvec{F}}}}_{{\varvec{r}}}$ [3]. This imbalance results in generation of the inertial effects characteristic of turbulent flow. In this case, the inertial effects exceed the dissipative, viscous effects. Additional energy input results in the generation of eddies, entities whose kinetic energy is larger than that of the surrounding fluid. During their movement and collision with other eddies they lose energy. When the kinetic energy of a single eddy become equal to the kinetic energy of the surrounding fluid, the flow attains local dynamical equilibrium and the eddy stops existing [3]. Consequently, the eddy lifetime represents the time necessary to dissipate the eddy’s energy. Larger eddies migrate faster and last longer [30].

**Fig. 1:**
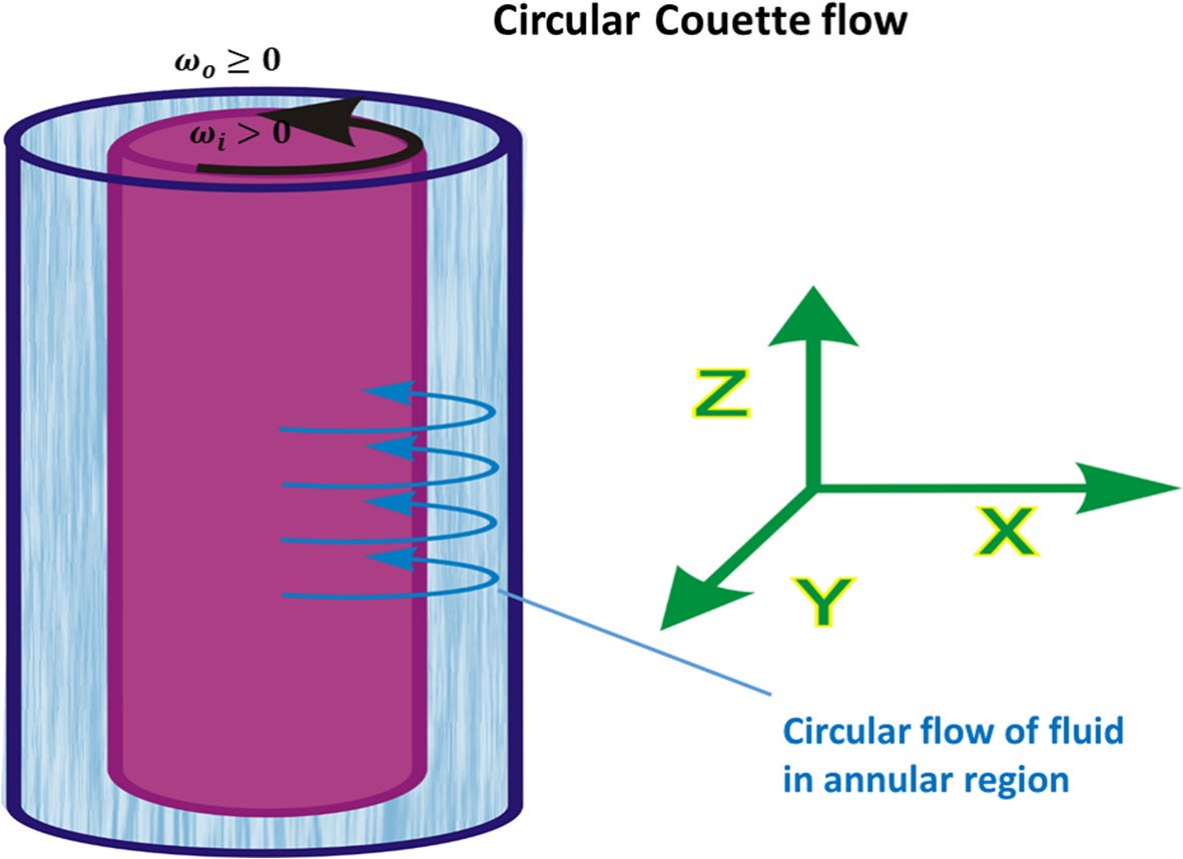
Schematic presentation of circular Couette flow

As the Reynolds number increases, Newtonian fluids pass through a range of intermediate flow states from laminar to turbulent. In the case of Couette flow this set of transient states, called the Taylor-Couette instabilities, has not extensively studied, even for Newtonian fluids [2]. The schematic presentation of circular Couette flow is shown in Fig. 1.

In the next section, we will discuss the elastic and elasto-inertial turbulence which occur at low and moderate ${R}_{e}$ number.

## Lower-${{\varvec{R}}}_{{\varvec{e}}}$ turbulence in polymer solutions

In lower-${R}_{e}$ turbulence, flow destabilisation is caused primarily by the viscoelastic behaviour of the systems [8, 15, 16]. In order to point to this cause-consequence relation, we will consider low and moderate-${R}_{e}$ turbulence in polymer solutions which behave as viscoelastic liquids, and in migrating epithelial collectives which behave as viscoelastic solids [8, 15, 16]. In the case of polymer solutions, besides the ${R}_{e}$ number, it is also necessary to introduce another dimensionless number to characterize the turbulence. It is the Weissenberg number which accounts for the polymer stress relaxation phenomenon. The moderate and high ${W}_{i}$ obtained during low-${R}_{e}$ Couette flow (for ${R}_{e}<1$) of polymer solutions was in the range of $1<{W}_{i}<\sim 30-50$ [16, 31]. As a further consideration, it is necessary to discuss the viscoelasticity of polymer solutions and to postulate their conservation of momentum and mass equations.

### Polymer solutions: their rheological behaviour

Despite its not physically correct, the Oldroyd-B constitutive model hasbecome the starting point for almost all complex flow calculations and analysis involving the behaviour of dilute polymer solutions [32]. The shear flow of these viscoelastic liquids induces generation of: (1) shear stress within the solvent ${\widetilde{{\varvec{\sigma}}}}_{{\varvec{l}}{\varvec{S}}}$ and (2) shear and normal stresses, ${\widetilde{{\varvec{\sigma}}}}_{{\varvec{p}}{\varvec{S}}}$ and ${\widetilde{{\varvec{\sigma}}}}_{{\varvec{p}}{\varvec{V}}}$ respectively, within the polymer chains. It is a common characteristic of viscoelastic systems that shear strain rate causes the generation of a volumetric strain rate (i.e. an extensional strain rate), resulting in the generation of both polymer shear and normal (tensional) stresses. The total stress within these systems can be expressed as the sum of these contributions: ${\widetilde{{\varvec{\sigma}}}}_{{\varvec{T}}}\left(r,t\right)={\widetilde{{\varvec{\sigma}}}}_{{\varvec{l}}{\varvec{S}}}+{\widetilde{{\varvec{\sigma}}}}_{{\varvec{p}}{\varvec{S}}}+{\widetilde{{\varvec{\sigma}}}}_{{\varvec{p}}{\varvec{V}}}$. The solvent behaves as a Newtonian fluid, while the polymer chains show viscoelastic behaviour caused primarily by inter- and intra-chain interactions. Consequently, the polymer stress includes viscous and elastic contributions, i.e. ${\widetilde{{\varvec{\sigma}}}}_{{\varvec{p}}{\varvec{k}}}={{\widetilde{{\varvec{\sigma}}}}_{{\varvec{p}}{\varvec{k}}}}^{{\varvec{v}}{\varvec{i}}{\varvec{s}}}+{{\widetilde{{\varvec{\sigma}}}}_{{\varvec{p}}{\varvec{k}}}}^{{\varvec{e}}{\varvec{l}}}$ (where $k\equiv S,V$, $S$ is shear, $V$ is volumetric, ${{\widetilde{{\varvec{\sigma}}}}_{{\varvec{p}}{\varvec{k}}}}^{{\varvec{v}}{\varvec{i}}{\varvec{s}}}$ is the viscous part of the polymer stress, and ${{\widetilde{{\varvec{\sigma}}}}_{{\varvec{p}}{\varvec{k}}}}^{{\varvec{e}}{\varvec{l}}}$ is the elastic part of the polymer stress). The rheological behaviour of the solvent corresponds to that of a Newtonian fluid and can be described by Eq. 1, while the rheological behaviour of the polymer chains can be described by the upper convected Maxwell model [15] expressed as:

3 $${\widetilde{{\varvec{\sigma}}}}_{{\varvec{p}}{\varvec{k}}}\left(r,t\right)+{\tau }_{Rpk}\frac{D{\widetilde{{\varvec{\sigma}}}}_{{\varvec{p}}{\varvec{k}}}}{Dt}={\upeta }_{pk}{\dot{\widetilde{{\varvec{\varepsilon}}}}}_{{\varvec{p}}{\varvec{k}}}\left(r,\tau \right)$$

where ${\tau }_{Rpk}$ is the polymer stress relaxation time, ${\upeta }_{pk}$ is the polymer viscosity (shear or bulk), $r$ is the space coordinate, ${\widetilde{{\varvec{\sigma}}}}_{{\varvec{p}}{\varvec{k}}}\left(r,t\right)$ is the polymer stress (normal or shear), $\frac{D{\widetilde{{\varvec{\sigma}}}}_{{\varvec{p}}{\varvec{k}}}}{Dt}$ are the material derivatives [1] expressed as $\frac{D{\widetilde{{\varvec{\sigma}}}}_{{\varvec{p}}{\varvec{k}}}}{Dt}=\frac{\partial {\widetilde{{\varvec{\sigma}}}}_{{\varvec{p}}{\varvec{k}}}}{\partial t}+({\overrightarrow{\nabla }{\overrightarrow{{\varvec{v}}}}_{{\varvec{p}}})}^{{\varvec{T}}}{\cdot \widetilde{{\varvec{\sigma}}}}_{{\varvec{p}}{\varvec{k}}}-{\widetilde{{\varvec{\sigma}}}}_{{\varvec{p}}{\varvec{k}}}\cdot \overrightarrow{\nabla }{\overrightarrow{{\varvec{v}}}}_{{\varvec{p}}}$**,** while ${\overrightarrow{{\varvec{v}}}}_{{\varvec{p}}}$ is the velocity of the polymers, ${\dot{\widetilde{{\varvec{\varepsilon}}}}}_{{\varvec{p}}{\varvec{k}}}\left(r,\tau \right)$ is the corresponding strain rate (shear or volumetric) such that ${\dot{\widetilde{{\varvec{\varepsilon}}}}}_{{\varvec{p}}{\varvec{S}}}\left(r,t\right)=\frac{1}{2}\left(\overrightarrow{\nabla }\overrightarrow{{{\varvec{v}}}_{{\varvec{p}}}}+{\overrightarrow{\nabla }\overrightarrow{{{\varvec{v}}}_{{\varvec{p}}}}}^{{\varvec{T}}}\right)$ is the shear strain rate, ${\dot{\widetilde{{\varvec{\varepsilon}}}}}_{{\varvec{p}}{\varvec{V}}}\left(r,t\right)=\overrightarrow{(\nabla }\cdot \overrightarrow{{{\varvec{v}}}_{{\varvec{p}}}})\widetilde{{\varvec{I}}}$ is the volumetric strain rate, $\widetilde{{\varvec{I}}}$ is the unit tensor. The main characteristics of the model proposed by Eq. 2 are: (1) polymer stress can relax under constant polymer strain rate conditions, (2) the strain rate and strain itself cannot relax, and (3) the stress change and the corresponding strain rate change occur on the same time scale [33]. The local system velocity $\overrightarrow{{\varvec{v}}}$ can be expressed as: $\overrightarrow{{\varvec{v}}}=\frac{{\rho }_{l}}{\rho }{\overrightarrow{{\varvec{v}}}}_{{\varvec{l}}}+\frac{{\rho }_{p}}{\rho }{\overrightarrow{{\varvec{v}}}}_{{\varvec{p}}}$ (where ${\rho }_{l}$ is the density of the solvent, ${\rho }_{p}$ is the polymer density, and $\rho$ is the density of the system $\rho ={\rho }_{l}+{\rho }_{p}$) [32]. The relationship between the normal stress generated within polymer chains under flow and the external shear strain rate can be expressed in the form of the first and second normal stress differences. The first normal stress difference for Couette flow ${N}_{1}$ is positive for the case of flexible polymer chains [34] and can be formulated as: ${N}_{1}={\sigma }_{p\theta \theta }-{\sigma }_{p rr}$ (where ${\sigma }_{p \theta \theta }$ and ${\sigma }_{p rr}$ are the components of polymer normal stress in cylindrical coordinates). During a shear flow, the polymer chains undergo stretching caused by extensional strain rate ${\dot{\widetilde{{\varvec{\varepsilon}}}}}_{{\varvec{p}}{\varvec{V}}}$. The stretching induces polymer local stiffening accompanied by a local increase in the normal stress within the polymer chains. In contrast, shear deformation has no effects on the polymer stiffness. Consequently, our attention is primarily directed to the flow conditions capable of altering the polymer stretching which can destabilise the flow. For example, in circular Couette flow, polymer stretching is pronounced due to generation of centrifugal force unlike the case of flow through pipes [20]. In Couette flow, the chain stretching occurs perpendicular to the flow direction.

The rapid increase in the polymer normal stress, caused by the chains stretching, is followed by stress relaxation. This relaxation toward the residual stress results in a relaxation of the polymer chains, which leads to the chain softening again. The relaxation of the normal polymer stress under constant extensional strain rate, i.e. ${\dot{\widetilde{{\varvec{\varepsilon}}}}}_{{\varvec{p}}{\varvec{V}}}={\text{const}}.$ can be expressed from Eq. 2 as:

4 $${\widetilde{{\varvec{\sigma}}}}_{{\varvec{p}}{\varvec{V}}}\left(r,t\right)={\widetilde{{\varvec{\sigma}}}}_{0{\varvec{p}}{\varvec{V}}}{e}^{-\frac{t}{{\tau }_{Rpk}}}+{\widetilde{{\varvec{\sigma}}}}_{{\varvec{r}}{\varvec{p}}{\varvec{V}}}\left(r\right)\left(1-{e}^{-\frac{t}{{\tau }_{RpV}}}\right)$$

where ${\widetilde{{\varvec{\sigma}}}}_{0{\varvec{p}}{\varvec{V}}}$ is the initial ‘’hoop stress’’ which includes the elastic and viscous contributions, i.e. ${\widetilde{{\varvec{\sigma}}}}_{0{\varvec{p}}{\varvec{V}}}={{\widetilde{{\varvec{\sigma}}}}_{0{\varvec{p}}{\varvec{V}}}}^{{\varvec{e}}{\varvec{l}}}+{{\widetilde{{\varvec{\sigma}}}}_{0{\varvec{p}}{\varvec{V}}}}^{{\varvec{v}}{\varvec{i}}{\varvec{s}}}$, while the polymer residual stress ${\widetilde{{\varvec{\sigma}}}}_{{\varvec{r}}{\varvec{p}}{\varvec{V}}}$ is purely viscous and dissipative, i.e. ${{\widetilde{{\varvec{\sigma}}}}_{{\varvec{r}}{\varvec{p}}{\varvec{V}}}=\eta }_{{\varvec{V}}}{\dot{\widetilde{{\varvec{\varepsilon}}}}}_{{\varvec{p}}{\varvec{V}}}$**.** The elastic contribution to the stress ${{\widetilde{{\varvec{\sigma}}}}_{0{\varvec{p}}{\varvec{V}}}}^{{\varvec{e}}{\varvec{l}}}$ can be about two orders of magnitude larger than the viscous contribution ${{\widetilde{{\varvec{\sigma}}}}_{0{\varvec{p}}{\varvec{V}}}}^{{\varvec{v}}{\varvec{i}}{\varvec{s}}}$ during low-${R}_{e}$ flow [16]. depending on: (1) the chain flexibility, (2) molecular weight, and (3) the magnitude of the chain stretching [32].

The elastic contribution to the polymer stress ${{\widetilde{{\varvec{\sigma}}}}_{0{\varvec{p}}{\varvec{V}}}}^{{\varvec{e}}{\varvec{l}}}$ decreases during the process of relaxation toward the equilibrium state, satisfying the condition that when $t\to {t}_{eq}$, the stress ${{\widetilde{{\varvec{\sigma}}}}_{0{\varvec{p}}{\varvec{V}}}}^{{\varvec{e}}{\varvec{l}}}\to 0$ and the viscous part of the stress is equal to the residual stress, i.e. ${{\widetilde{{\varvec{\sigma}}}}_{0{\varvec{p}}{\varvec{V}}}}^{{\varvec{v}}{\varvec{i}}{\varvec{s}}}\approx {\widetilde{{\varvec{\sigma}}}}_{{\varvec{r}}{\varvec{p}}{\varvec{V}}}$ (where ${t}_{eq}$ is the time for reaching the equilibrium state). The existence of eddies in the flow of polymer solutions is related to polymer local stiffening. Consequently, the lifetime of the eddies ${t}_{LT}$ in this case corresponds to the polymer stress relaxation time, which is included in the dimensionless ${W}_{i}$ number. Consequently, the lifetime of the eddies is ${t}_{LT}\sim {\tau }_{RpV}.$ This rapid increase in the elastic contribution to the polymer stress caused by the polymer stretching is the main cause of the flow destabilisation and could result in the generation of the inertial effects. In this context, two variants of the phenomenon have been discussed: (1) elastic turbulence and (2) elastic-inertial turbulence [15, 20]. The existence of inertial effects in elastic turbulence depends on the inter-relation between driving and resistive forces.

### Conservation of momentum and mass equations for the flow of polymer solutions

**Fig. 2:**
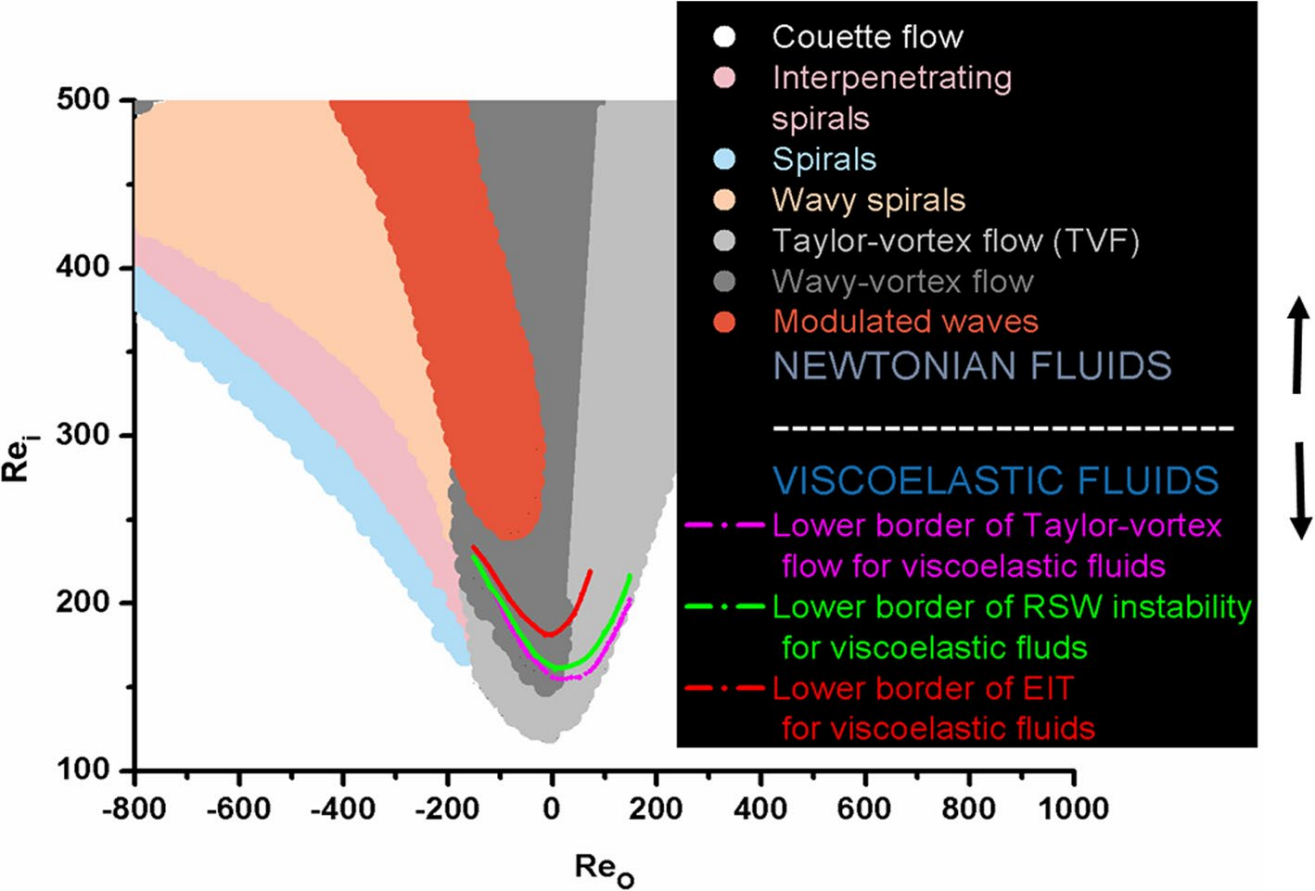
The phase diagram for Taylor-Couette flow of two different fluids: viscoelastic polymer solutions (shown as regions between coloured lines) and Newtonian fluids (represented as coloured regions); The phase diagram that was developed was influenced by sources [2, 20]

The flow of polymer solutions can be treated as incompressible, i.e. $\overrightarrow{\nabla }\cdot \overrightarrow{{\varvec{v}}}=0$. Similarly, to the previous case, the flow of polymer solutions can be expressed as a conservation of momentum, inspired by the experimental findings of Groisman and Steinberg [15] and Li and Steinberg [35], as:

5 $$\rho \frac{D\overrightarrow{{\varvec{v}}}}{Dt}={\overrightarrow{{\varvec{F}}}}_{{\varvec{d}}}-{\overrightarrow{{\varvec{F}}}}_{{\varvec{v}}{\varvec{i}}{\varvec{s}}}-{\overrightarrow{{\varvec{F}}}}_{{\varvec{f}}{\varvec{r}}}$$

where $\frac{D\overrightarrow{{\varvec{v}}}}{Dt}=\frac{\partial \overrightarrow{{\varvec{v}}}}{\partial t}+(\overrightarrow{{\varvec{v}}}\cdot \overrightarrow{\nabla })\overrightarrow{{\varvec{v}}}$ is the material derivatives [1], $\rho$ is the polymer solution density and $\overrightarrow{{\varvec{v}}}$ is the local velocity of the solution. The driving force is ${\overrightarrow{{\varvec{F}}}}_{{\varvec{d}}}=-\overrightarrow{\nabla }p$, while the resistive force includes two contributions, the viscoelastic force ${\overrightarrow{{\varvec{F}}}}_{{\varvec{v}}{\varvec{i}}{\varvec{s}}}=\nabla \cdot {{({{\widetilde{{\varvec{\sigma}}}}_{{\varvec{p}}{\varvec{V}}}}^{{\varvec{e}}{\varvec{l}}}+\widetilde{{\varvec{\sigma}}}}_{{\varvec{p}}{\varvec{V}}}}^{{\varvec{v}}{\varvec{i}}{\varvec{s}}}+{\widetilde{{\varvec{\sigma}}}}_{{\varvec{l}}{\varvec{S}}})$ and the frictional force ${\overrightarrow{{\varvec{F}}}}_{{\varvec{f}}{\varvec{r}}}=\rho {\xi }_{eff}\overrightarrow{{\varvec{v}}}$. Here ${\xi }_{eff}$ is the effective frictional coefficient caused by solvent flow past polymer chains expressed as ${\xi }_{eff}=\frac{1}{2}{C}_{d}\rho \Vert \overrightarrow{{\varvec{v}}}\Vert {r}_{H}^{2}$, ${C}_{d}$ is the dimensionless drag coefficient, $\Vert \overrightarrow{{\varvec{v}}}\Vert$ is the solution speed, and ${r}_{H}$ is the hydraulic radius of the polymer cluster. The effective friction coefficient depends on inter- and intra-chain interactions which are responsible for the chain stiffening and can be expressed as ${\xi }_{eff}={\xi }_{eff}\left({{\widetilde{{\varvec{\sigma}}}}_{{\varvec{p}}{\varvec{V}}}}^{{\varvec{e}}{\varvec{l}}}\right)$. Li and Steinberg [35] revealed that the drag coefficient increases with the dimensionless ${W}_{i}$ number during the low-${R}_{e}$ flow of the solution through a channel. The main question is: can an increase in the effective friction, caused by the stiffening of the polymer chains, induce a perturbation of the dynamical force equilibrium such that ${\overrightarrow{{\varvec{F}}}}_{{\varvec{d}}}-{\overrightarrow{{\varvec{F}}}}_{{\varvec{v}}{\varvec{i}}{\varvec{s}}}-{\overrightarrow{{\varvec{F}}}}_{{\varvec{f}}{\varvec{r}}}\ne 0$? This could be happening in circular Couette flow, caused by the work of the centrifugal force, rather than during the solution flow through pipes/channels depending on the polymer’s flexibility, concentration and molecular weight [36]. If it arose, this type of turbulence would correspond to low (or moderate) -${R}_{e}$ elasto-inertial turbulence. Otherwise, it would be elastically induced unstable flow, declared as low-${R}_{e}$ elastic turbulence (known as an inertia-less turbulence [37]), representing some transient, chaotic, state arising during the development of turbulent flow [20]. Three types of instability have been discussed: shear-dominated, extensional-dominated, and mixed instabilities [12]. The elasto-inertial turbulence has been recognised during Couette flow of polyethylene oxide solutions for ${R}_{ei}\ge 200$ and ${R}_{eo}=0$ (where ${R}_{ei}$ is the Reynolds number of the inner cylinder and ${R}_{eo}$ is the Reynolds number of the outer cylinder) [20]. For the case of Couette flow of Newtonian fluids, turbulence occurs for ${R}_{ei}\ge 1500$ and ${R}_{eo}=0$ [2]. The phase diagram for Taylor-Couette flow of Newtonian fluids and viscoelastic polymer solutions is shown in Fig. 2.

Interestingly, while polymer chains increase the friction coefficient during the low-${R}_{e}$ flow, they exert an opposite effect on the friction coefficient under the high-${R}_{e}$ flow of the solution. Tabor and De Gennes [38] pointed out that when the kinetic energy of polymer chains becomes equal to the elastic energy, the chains act to reduce the friction coefficient by perturbing the turbulent cascade, i.e. the distribution of kinetic energy. This phenomenon can appear during the flow of polymer solutions through pipes for ${R}_{e}>50000$ [39].

In further consideration, we will compare the low-${R}_{e}$ elasto-inertial turbulence arising during the flow of polymer solutions with the low-${R}_{e}$ turbulence, i.e. the active turbulence, recognized during collective cell migration.

## Low-${{\varvec{R}}}_{{\varvec{e}}}$ turbulence in migrating epithelial collectives

While viscoelastic polymer solutions need an external force to initiate the flow, multicellular systems self-generate energy for cellular migration and follow extracellular signals [17, 40]. Directional cell movement, i.e. taxis can be induced by various chemical, mechanical, and electrical stimuli. Consequently, established gradient of: (1) soluble chemical cues induces chemotaxis, (2) an electric field induces galvanotaxis, (3) the stiffness of a substrate matrix or adjacent tissue induces durotaxis, and (4) cellular adhesion sites or substrate-bound cytokines induce haptotaxis [40, 41]. Consequently, this form of low-${R}_{e}$ turbulence is caused by an inhomogeneous distribution of stored elastic energy rather than by kinetic energy. Butler et al. [42] calculated the strain energy of a single muscle cell adhering to a substrate from the work of the traction force. The corresponding cell strain energy is $\sim {10}^{-12} {\text{J}}$. The average kinetic energy is the smallest energetic part for single cell and equal to $\sim {10}^{-28} {\text{J}}$ (for corresponding cell speed $1 \frac{\mu m}{min}$ and average single cell mass of $\sim {10}^{-12}\mathrm{ kg}$ [43].

Low-${R}_{e}$ turbulence in migrating epithelial collectives in the form of propagating and standing waves has been recognised in several model systems [6–8, 21, 25, 26]. The propagating and standing waves are the consequence of long-time inertial effects [7, 8]. While the standing waves are generated during the confluent cell monolayers which can result in the cell swirling motion, the propagating waves have been recognised during free expansion of epithelial monolayers [6]. The long-time inertial effects are related to oscillations in cell velocity, and in the corresponding strain and resultant cell stress [6, 7, 25, 26], as well as to the geometrical characteristics of multicellular systems [23, 25, 27]. Some authors have discussed low-${R}_{e}$ turbulence of multicellular systems in the context of effective inertia [7, 8, 44], while some of them neglect inertial effects [6]. Banerjee et al. [44] and Notbohm et al. [7] considered the effective inertia as a product of the viscoelasticity of multicellular system by considering the phenomenon on a cellular level. Notbohm et al. [7] emphasized that the effective inertia arises as a result of coupling between cell contractility and strain, in the form of cell active stress which depends on the myosin concentration. Banerjee et al. [44] coupled local strain with cell contractility and polarization. Pajic-Lijakovic and Milivojevic [8] discussed the appearance of effective inertia as a product of the system viscoelasticity by considering the phenomenon on a supracellular level. Serra-Picamal et al. [6] neglected long-time inertial effects. They postulated that the cytoskeletal reinforcement and fluidization result in a biphasic stress response in single cells which are responsible for generation of mechanical waves. Deforet et al. [45] simulated cell rearrangement caused by collective cell migration and included the effective inertia into the conservation of momentum equation formulated at cellular level.

The main characteristic of collective cell migration is its multi-time-scale nature [8, 46]. A short time-scale (i.e. a time scale of minutes) corresponds to the remodelling of cell–cell and cell–matrix adhesion contacts, while a long time-scale (i.e. a time-scale of hours) corresponds to cell movement. Cell division can be neglected because it occurs on a time-scale of days. The average doubling time of epithelial MCF-10A cells is $\sim 1 {\text{day}}$ [47], for human keratinocytes it is $\sim 2-3 {\text{days}}$ [48], and for MDCK cells it is $\sim 5 {\text{days}}$ [49]. This doubling time may increase further under adverse conditions, such as a rise in the cell packing density ${n}_{e}$.

### The main causes of the generation of the Low-${{\varvec{R}}}_{{\varvec{e}}}$ turbulence in cellular systems

Collective cell migration generates cell mechanical stress [6, 7]. Epithelial cells have developed biological mechanisms to respond to this mechanical stress and even to regulate it. These mechanisms account for the interplay between biological processes such as: remodelling of cell–cell and cell–matrix adhesion contacts; contact inhibition of locomotion (CIL); cell polarity alignment; the cell-jamming state transition; and the epithelial-to-mesenchymal cell state transition (EMT) [17, 18, 50, 51]. The remodelling of cell–cell and cell–matrix adhesion contacts is related to the gene expression depending on micro-environmental conditions [17, 52]. The CIL represents cell head-to-head collisions, which are intensive for higher cell packing densities, which can induce weakening of the cell–cell adhesion contacts and which can down-regulate cell propulsion [53].

**Fig. 3:**
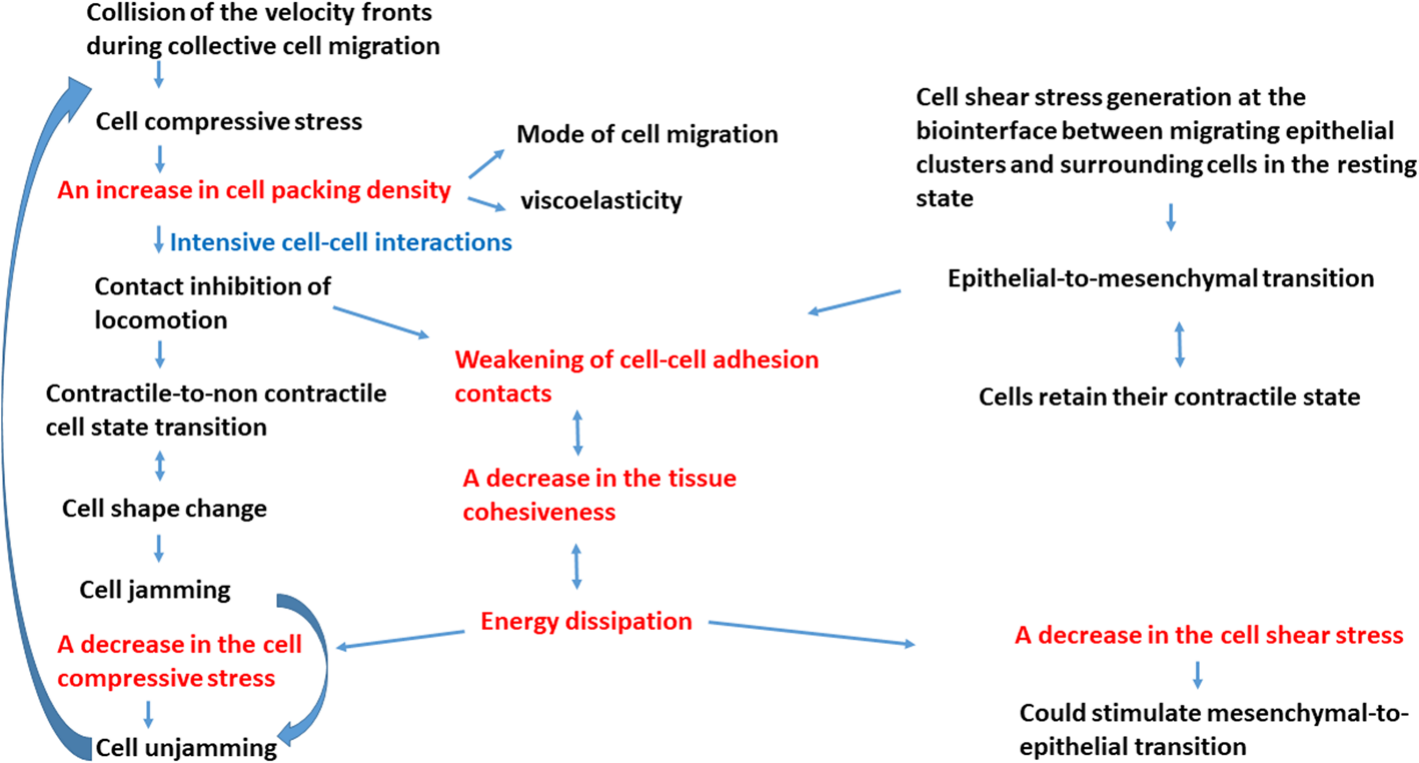
Schematic representation of cell response under mechanical stress caused by collective cell migration

The main characteristic of migrating epithelial collectives is the inhomogeneous distribution of cell packing density and cell velocity [6, 28, 54, 55]. Tlili et al. [55] considered the free expansion of Madin-Darby canine kidney type II (MDCK) epithelial monolayers and revealed that cell packing density varies from ${1x10}^{5} \frac{{\text{cells}}}{{{\text{cm}}}^{2}}$ to ${5x10}^{5} \frac{{\text{cells}}}{{{\text{cm}}}^{2}}$. An increase in cell packing density from ${1x10}^{5} \frac{{\text{cells}}}{{{\text{cm}}}^{2}}$ to ${5x10}^{5} \frac{{\text{cells}}}{{{\text{cm}}}^{2}}$ resulted in a decrease in cell velocity from $0.8 \frac{\mathrm{\mu m}}{{\text{min}}}$ to zero [55]. Nnetu et al. [54] considered the free expansion of epithelial MCF-10A cell monolayers and pointed out that cell velocity drops to zero at a cell packing density of $\sim {3.5x10}^{5} \frac{{\text{cells}}}{{{\text{cm}}}^{2}}$. corresponding to cell-jamming. This cell packing density was correlated with the cell mechanical stress caused by collective cell migration [56]. The cell-jamming state transition, which leads to a separation of the system’s contractile and non-contractile states, is caused by inter-dependent processes such as: (1) an increase in cell packing density [28, 54], (2) change in the strength of cell–cell adhesion contacts [57, 58], (3) the magnitude of cellular forces and persistence time for these forces [58], (4) cell shape changes [57], and (5) contact inhibition of locomotion (CIL) [59]. Higher cell packing density, characteristic of the jammed state, leads to CIL, which results in a weakening of the cell–cell adhesion contacts. The contractile to non-contractile cell state transition also influences the shapes of single cells [57]. Under jamming, the cells enter a non-contractile, resting state resulting in a softening of the multicellular system. Schulze et al. [60] revealed that the Young’s modulus of contractile MDCK cell monolayer is ~ 33.0 ± 3.0 kPa, while the modulus of non-contractile cells is approximately twice lower. The weakening of cell–cell adhesion contacts causes energy dissipation and a decrease in the cell compressive stress. A decrease in the compressive stress leads to cell-unjamming. Then the cells establish the contractile state and strong cell–cell adhesion contacts and start migration again. Consequently, the cell jamming-to-unjamming transition is the cell mechanism applied to regulate the compressive stress [51]. A schematic presentation of the cell response under mechanical stress caused by collective cell migration is shown in Fig. 3.

While cells well tolerate a normal cell stress of a few hundreds of Pa, a shear stress of a few tens of Pa is enough to induce an inflammation of epithelial collectives and even cell death [61–64]. Cell response to the shear stress generated during collective cell migration is primarily related to the epithelial-to-mesenchymal transition (EMT). While a cell compressive stress of a few hundred Pa can also induce the EMT, this process can be initiated under very low shear stress of $<1 {\text{Pa}}$ [65]. The main characteristics of epithelial-like cells are their: cuboidal shape, reduced cell mobility, and apical-basal polarity, and the establishment of strong E-cadherin-mediated cell–cell adhesions. In contrast, mesenchymal-like cells can be characterized by their: elongated shape, increased migratory cell ability, establishment of front-rear cell polarity, and weak N-cadherin-mediated cell–cell adhesion [66]. The EMT is a process in which cells decrease their cohesiveness leading to: (1) energy dissipation accompanied by a decrease in the shear stress and (2) establishment of a cell swirling motion caused by the work done by shear stress torque [9]. The cell swirling motion is a way for cells to reduce single cell exposure to the undesirable shear stress. It is known that the local shear strain within the swirl is lower than the macroscopic shear strain established in the surroundings of the swirl [9].

Both processes, the jamming state transition and the EMT have a feedback on the cell speed and viscoelasticity accompanied by the generation of mechanical stress (both shear and normal). In order to understand the low-${R}_{e}$ turbulence in multicellular systems, it is necessary to discuss the rheological behaviour and surface characteristics of multicellular systems in more detail.

### Epithelial multicellular systems: rheological behaviour

**Fig. 4:**
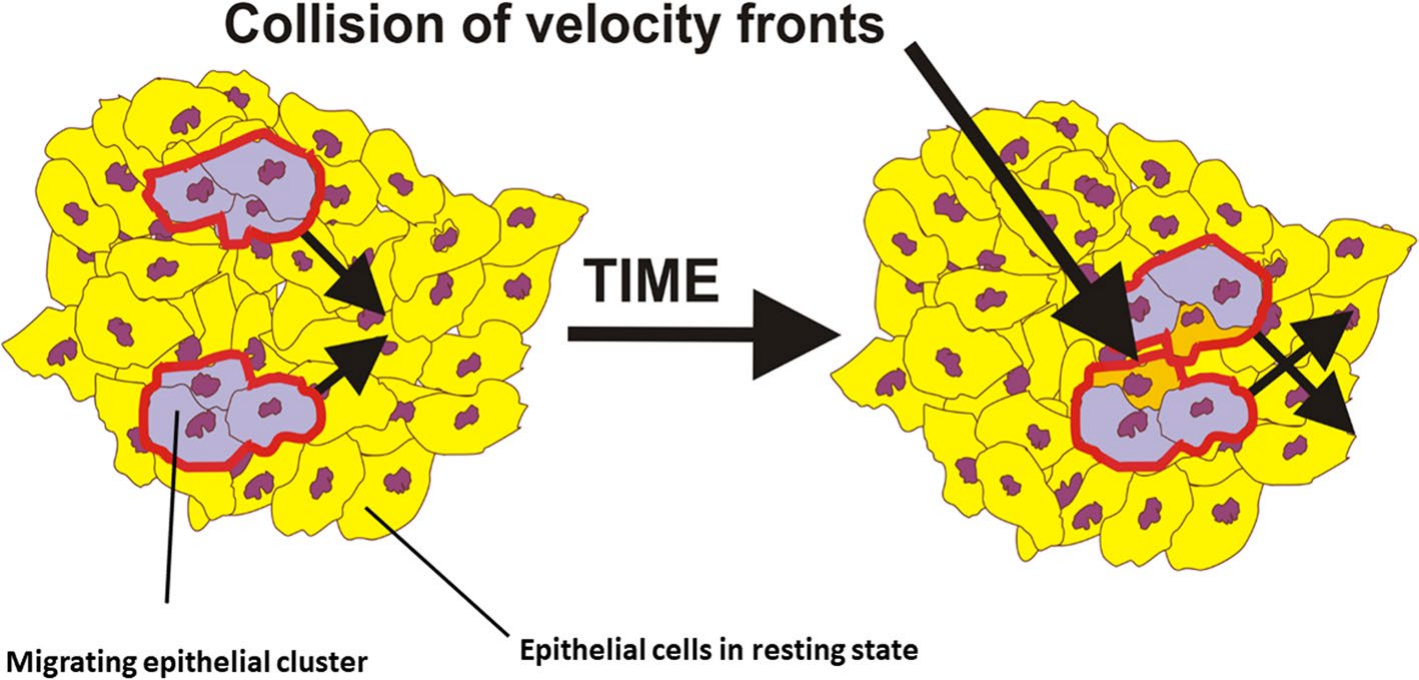
Schematic presentation of the collision of two cell velocity fronts during collective cell migration

The ‘’flow’’ of multicellular systems caused by collective cell migration is primarily extensional, while compressional and shear strains also exist locally [6, 7, 33]. Extension in one direction results in compression in the other directions in order to preserve the structural integrity of multicellular systems [8]. These strains generate cell stresses. The viscoelasticity of multicellular systems caused by collective cell migration exhibits several important characteristics, including:

- The cell mechanical stress can have both normal (tensional/compressive) and shear components [6, 67]. The tensional stress is generated during the free expansion of cell monolayers, while compressive stress is primarily induced by the collision of local forward and backward flows [6, 51]. Compressive stress is also generated within the cellular systems under higher cell packing density such as: (1) the rearrangement of confluent cell monolayers [7] and (2) the collision of oppositely directed velocity fronts at the contact point between two cell aggregates during their fusion as was shown in Fig. 4 [23].
- Shear stress is generated along the biointerface between migrating epithelial clusters and surrounding cells in the resting state [62].
- The ability of the cell stress to relax depends on the cell packing density. For a cell packing density ${n}_{e}\le {n}_{conf}$ (where ${n}_{conf}$ is the cell packing density at the confluent state), the stress can relax. Petitjean et al. [68] pointed out that the MDCK cell monolayers reached the confluence for a cell packing density of ${n}_{conf}\sim 2.5x{10}^{5} \frac{{\text{cells}}}{{{\text{cm}}}^{2}}$ and a cell velocity of $\sim 0.14 \frac{\mathrm{\mu m}}{{\text{min}}}$.
- An increase in the cell packing density changes the state of viscoelasticity. For higher cell packing density, which corresponds to a cell state near jamming, the stress cannot relax. Consequently, three mechanisms of cell migration can be considered depending on the cell packing density: (1) a convective mechanism for ${n}_{e}\le {n}_{conf}$, (2) a diffusion mechanism for ${n}_{j}>{n}_{e}>{n}_{conf}$ (where ${n}_{j}$ is the cell packing density under the jamming state), and (3) a sub-diffusive mechanism for ${n}_{e}\to {n}_{j}$.
- When the cell stress can relax toward the cell residual stress, the process is exponential [46, 69] and occurs on a time-scale of minutes [46, 69]. The stress relaxation is primarily caused by the remodelling of cell–cell adhesion contacts and cell shape changes [8, 46].
- The cell strain can relax under constant stress conditions. Relaxation occurs via collective cell migration on a time-scale of hours [46].
- The epithelial stress relaxes within many short-time relaxation cycles under constant strain per cycle, while the strain change occurs on a time-scale of hours [8].
- The cell residual stress remains in the system in the absence of external forces. It is elastic when epithelial cells retain their phenotype for the condition that ${n}_{e}\le {n}_{conf}$. It is in accordance with fact that the epithelial residual stress correlates with the corresponding strain during free expansion of epithelial monolayers; the rearrangement of confluent epithelial monolayers has been confirmed experimentally [6, 7]. When the epithelial cells undergo the epithelial-to-mesenchymal transition, the corresponding residual stress becomes purely dissipative due to weakening of the cell–cell adhesion contacts.
- Cell residual stress accompanied by the corresponding strain and cell velocity oscillates on a time-scale of hours [6–8]. This phenomenon, described as a mechanical waves, represents a part of the low-${R}_{e}$ turbulence. A description of the main characteristics of this type of turbulence will be given in more detail below.
- An externally-applied compressive or shear stress reduces the movement of epithelial cells. A compressive stress of 773 Pa suppresses the movement of epithelial breast MCF-10A and MCF-7 cells [70]. A shear stress of only 1.5 Pa reduces movement of MCF-10A cells [71].

In further consideration, we will discuss the phenomenon of low-${R}_{e}$ turbulence in relation to two epithelial model systems: (1) the fusion of two epithelial aggregates and (2) the free extension of epithelial monolayers on a substrate matrix. Epithelial cells within cell aggregates only establish cell–cell adhesion contacts, whereas epithelial cells within monolayers establish both, cell–cell and cell–matrix adhesion contacts.

The cell normal residual stress, generated within migrating epithelial collectives, has been expressed as [72]:

6 $${\widetilde{{\varvec{\sigma}}}}_{{\varvec{e}}{\varvec{r}}{\varvec{V}}}=+\Delta \boldsymbol{p}_{c}\widetilde{{\varvec{I}}}+{{\widetilde{{\varvec{\sigma}}}}_{{\varvec{e}}{\varvec{r}}{\varvec{V}}}}^{{\varvec{C}}{\varvec{C}}{\varvec{M}}}$$

where ${\widetilde{{\varvec{\sigma}}}}_{{\varvec{e}}{\varvec{r}}{\varvec{V}}}$ is the normal residual stress within the epithelium, $\Delta {p}_{c}$ is the isotropic part of the cell residual stress, while ${{\widetilde{{\varvec{\sigma}}}}_{{\varvec{e}}{\varvec{r}}{\varvec{V}}}}^{{\varvec{C}}{\varvec{C}}{\varvec{M}}}$ is the deviatoric part of the residual stress caused by collective cell migration, and $\widetilde{{\varvec{I}}}$ is the unity tensor. The isotropic part of the cell normal stress is:

1. $\Delta {p}_{c}=-{\gamma }_{e }\left(\overrightarrow{\nabla }\cdot \overrightarrow{{\varvec{n}}}\right)$ for the fusion of two epithelial aggregates (where ${\gamma }_{e}$ is the epithelial surface tension and $\overrightarrow{{\varvec{n}}}$ is the normal vector to the aggregate surface), or
2. $\Delta {p}_{c}=-{\gamma }_{em }\left(\overrightarrow{\nabla }\cdot \overrightarrow{{\varvec{n}}}\right)$ for movement of epithelial monolayers on a substrate matrix (where ${\gamma }_{em}$ is the epithelial-matrix interfacial tension).

When the cell rearrangement during the fusion of two cell aggregates is discussed, it is necessary to take into consideration the change in epithelial surface tension, while the extracellular matrix is not presented in this model system. Cells establish only the cell–cell adhesion contacts. The epithelial surface tension ${\gamma }_{e}$ is a time-dependent physical parameter quantifying the energy of a multicellular surface in contact with a liquid medium, i.e. the surface cohesion. This parameter depends on: the strength of E-cadherin-mediated cell–cell adhesion contacts, the cell contractility, and the surface deformability [24]. The cell contractility enhances the strength of E-cadherin-mediated cell–cell adhesion contacts leading to an increase in the epithelial surface tension [73]. The extension of a multicellular surface, caused by collective cell migration, results in an increase in the epithelial surface tension [74]. The main characteristic of the latter is an inhomogeneous distribution along the multicellular surface, caused primarily by a remodelling of the cell–cell adhesion contacts under local cell strain.

When the movement of cell monolayers on a substrate matrix is discussed, it is necessary to take into account changes in the epithelial-matrix interfacial tension ${\gamma }_{em}$ and in the matrix surface tension ${\gamma }_{m}$, as well as the epithelial surface tension ${\gamma }_{e}$. While the epithelial surface tension has been measured under simplified conditions (i.e. the equilibrium, static epithelial surface tension) [23, 75], the epithelial-matrix interfacial tension has not yet been measured. The epithelial-matrix interfacial tension ${\gamma }_{em}$ and the matrix surface tension are also time-dependent parameters. The migrating epithelial cells, in this case, exert traction on the surface matrix by altering the matrix rearrangement, which has a feedback impact on the matrix surface tension ${\gamma }_{m}$. The epithelial-matrix interfacial tension can be expressed as ${\gamma }_{em }={\gamma }_{e }+{\gamma }_{m}-{e}_{a}$ (where ${\gamma }_{m}$ is the surface tension of the substrate matrix and ${e}_{a}$ is the adhesion energy between the epithelial cells and matrix per unit area of the biointerface equal to ${e}_{a}={\rho }_{e-m}\frac{1}{2}k{\left|{\overrightarrow{{\varvec{u}}}}_{{\varvec{m}}}\right|}^{2}$, ${\rho }_{e-m}$ is the surface packing density of cell–matrix adhesion contacts, k is the spring constant of the single focal adhesion and ${\overrightarrow{{\varvec{u}}}}_{{\varvec{m}}}$ is the matrix displacement field [76]). When epithelial cells establish stronger cell–matrix focal adhesions, the interfacial tension is lower. The main characteristic of the epithelial-matrix interfacial tension is its inhomogeneous distribution along the epithelial-matrix biointerface.

An inhomogeneous distribution of epithelial surface tension (for the fusion of two cell aggregates) generates a cell shear stress along the multicellular surface of the cell aggregates. Similarly, an inhomogeneous distribution of the epithelial-matrix interfacial tension (for the migration of cell monolayers on a substrate matrix) generates cell shear stress along the epithelial-matrix biointerface. In both cases, the shear stress is induced by cell movement from the region of lower surface tension/interfacial tension to the region of larger surface tension/interfacial tension along the surface/biointerface. Gsell et al. [77] recently confirmed cell movement along the multicellular surface in contact with a liquid medium, driven by the gradient in tissue surface tension. This is a part of the Marangoni effect. The latter has been recognized in various soft matter systems caused by temperature or concentration gradients [78]. The other part of the cell shear stress is induced by collective cell migration.

Consequently, the shear stress generated along the surface of cell aggregates, caused by the cell aggregate fusion, can be written:

7 $$\overrightarrow{{\varvec{n}}}\cdot {\widetilde{{\varvec{\sigma}}}}_{{\varvec{e}}{\varvec{r}}{\varvec{S}}}\cdot \overrightarrow{{\varvec{t}}}={\overrightarrow{\nabla }}_{{\varvec{s}}}{\gamma }_{e}\cdot \overrightarrow{{\varvec{t}}}+\overrightarrow{{\varvec{n}}}\cdot {{\widetilde{{\varvec{\sigma}}}}_{{\varvec{e}}{\varvec{r}}{\varvec{S}}}}^{\boldsymbol{CCM}}\cdot \overrightarrow{{\varvec{t}}}.$$

where ${\widetilde{{\varvec{\sigma}}}}_{{\varvec{e}}{\varvec{r}}{\varvec{S}}}$ is the total shear stress, $\overrightarrow{\nabla }{\gamma }_{e}$ is the gradient of epithelial surface tension, ${{\widetilde{{\varvec{\sigma}}}}_{{\varvec{e}}{\varvec{r}}{\varvec{S}}}}^{CCM}$ is the shear stress caused by collective cell migration, and $\overrightarrow{{\varvec{t}}}$ is a vector tangent to the aggregate surface. The shear stress generated along the epithelial-matrix biointerface, caused by the migration of epithelial monolayers on a substrate matrix, can be written:

8 $$\overrightarrow{{\varvec{n}}}\cdot {\widetilde{{\varvec{\sigma}}}}_{{\varvec{e}}{\varvec{r}}{\varvec{S}}}\cdot \overrightarrow{{\varvec{t}}}={\overrightarrow{\nabla }}_{{\varvec{s}}}{\gamma }_{em}\cdot \overrightarrow{{\varvec{t}}}+\overrightarrow{{\varvec{n}}}\cdot {{\widetilde{{\varvec{\sigma}}}}_{{\varvec{e}}{\varvec{r}}{\varvec{S}}}}^{\boldsymbol{CCM}}\cdot \overrightarrow{{\varvec{t}}}.$$

where ${{\overrightarrow{\nabla }}_{{\varvec{s}}}\gamma }_{em}$ is the gradient of the epithelial-matrix interfacial tension. The total cell residual stress is: ${\widetilde{{\varvec{\sigma}}}}_{{\varvec{e}}{\varvec{r}}{\varvec{T}}}={\widetilde{{\varvec{\sigma}}}}_{{\varvec{e}}{\varvec{r}}{\varvec{V}}}+{\widetilde{{\varvec{\sigma}}}}_{{\varvec{e}}{\varvec{r}}{\varvec{S}}}$**.**

The cell stress (normal and shear), caused by collective cell migration, depends on the cell packing density which is inhomogeneously distributed within epithelial systems [28, 79]. The proposed constitutive models depending on the cell packing density and cell speed are presented in Table 1.

**Table 1:** Viscoelasticity of migrating epithelial collectives

Characteristics of cell movement	Cell packing density Cell speed	Constitutive model
Convective cell migration	${n}_{e}\le {n}_{conf}$ $0.1 <\Vert {\overrightarrow{{\varvec{v}}}}_{{\varvec{e}}}\Vert <\sim 1 \frac{\mu m}{min}$	The Zener model for viscoelastic solids: ${{\widetilde{{\varvec{\sigma}}}}_{{\varvec{e}}{\varvec{k}}}}^{CCM}\left(r,t,\tau \right)+{\tau }_{Rck} {{\dot{\widetilde{{\varvec{\sigma}}}}}_{{\varvec{e}}{\varvec{k}}}}^{CCM}={E}_{ck}{\widetilde{{\varvec{\varepsilon}}}}_{{\varvec{e}}{\varvec{k}}}\left(r,\tau \right)+{\eta }_{ck}{\dot{\widetilde{{\varvec{\varepsilon}}}}}_{{\varvec{e}}{\varvec{k}}}$ Stress relaxation under constant strain condition ${\widetilde{{\varvec{\varepsilon}}}}_{0{\varvec{c}}{\varvec{k}}}$ per single short-time relaxation cycle: ${{\widetilde{{\varvec{\sigma}}}}_{{\varvec{e}}{\varvec{k}}}}^{CCM}\left(r,t,\tau \right)={\widetilde{{\varvec{\sigma}}}}_{0{\varvec{e}}{\varvec{k}}}{e}^{-\frac{t}{{\tau }_{Rck}}}+{{\widetilde{{\varvec{\sigma}}}}_{{\varvec{r}}{\varvec{e}}{\varvec{k}}}}^{CCM}\left(r,\tau \right)\left(1-{e}^{-\frac{t}{{\tau }_{Rck}}}\right)$ Cell residual stress is elastic. ${{\widetilde{{\varvec{\sigma}}}}_{{\varvec{r}}{\varvec{e}}{\varvec{k}}}}^{CCM}={E}_{ck} {\widetilde{{\varvec{\varepsilon}}}}_{{\varvec{e}}{\varvec{k}}}$
Conductive (diffusion) cell migration	${n}_{j}>{n}_{e}>{n}_{conf}$ ${n}_{j}$ is the cell packing density at the jamming state $\Vert {\overrightarrow{{\varvec{v}}}}_{{\varvec{e}}}\Vert \sim {10}^{-3}-{10}^{-2}\frac{\mu m}{min}$	The Kelvin-Voigt model for viscoelastic solids: ${{\widetilde{{\varvec{\sigma}}}}_{{\varvec{e}}{\varvec{k}}}}^{CCM}\left(r,\tau \right)={E}_{ck}{\widetilde{{\varvec{\varepsilon}}}}_{{\varvec{e}}{\varvec{k}}}+{{\eta }_{ck} \dot{\widetilde{{\varvec{\varepsilon}}}}}_{{\varvec{e}}{\varvec{k}}}$ The stress cannot relax. ${{\widetilde{{\varvec{\sigma}}}}_{{\varvec{e}}{\varvec{k}}}}^{CCM}={{\widetilde{{\varvec{\sigma}}}}_{{\varvec{r}}{\varvec{e}}{\varvec{k}}}}^{CCM}$ A long-time change of the stress accounts for elastic and viscous contributions.
Damped conductive (sub-diffusion) cell migration near the cell jamming	${n}_{e}\to {n}_{j}$ $\Vert {\overrightarrow{{\varvec{v}}}}_{{\varvec{e}}}\Vert \to 0$	The Fraction model for the jamming state for viscoelastic solids: ${{\widetilde{{\varvec{\sigma}}}}_{{\varvec{e}}{\varvec{k}}}}^{CCM}\left(r,\tau \right)={\upeta }_{\alpha k}{D}^{\alpha }\left({\widetilde{{\varvec{\varepsilon}}}}_{{\varvec{e}}{\varvec{k}}}\right)$ For $0<\alpha <1/2$ The stress cannot relax. ${{\widetilde{{\varvec{\sigma}}}}_{{\varvec{e}}{\varvec{k}}}}^{CCM}={{\widetilde{{\varvec{\sigma}}}}_{{\varvec{r}}{\varvec{e}}{\varvec{k}}}}^{CCM}$
Convective cell migration after epithelial-to-mesenchymal cell state transition	${n}_{e}\le {n}_{conf}$ $\Vert {\overrightarrow{{\varvec{v}}}}_{{\varvec{e}}}\Vert \ge 1 \frac{\mu m}{min}$	The Maxwell model for viscoelastic liquids: ${{\widetilde{{\varvec{\sigma}}}}_{{\varvec{e}}{\varvec{k}}}}^{CCM}\left(r,t,\tau \right)+{\tau }_{Rck}{{\dot{\widetilde{{\varvec{\sigma}}}}}_{{\varvec{e}}{\varvec{k}}}}^{{\varvec{C}}{\varvec{C}}{\varvec{M}}}={\upeta }_{ck}{\dot{\widetilde{{\varvec{\varepsilon}}}}}_{{\varvec{e}}{\varvec{k}}}\left(r,\tau \right)$ Stress relaxation under constant strain rate ${\dot{\widetilde{{\varvec{\varepsilon}}}}}_{0{\varvec{c}}{\varvec{k}}}$ per single short-time relaxation cycle: ${{\widetilde{{\varvec{\sigma}}}}_{{\varvec{e}}{\varvec{k}}}}^{CCM}\left(r,t,\tau \right)={\widetilde{{\varvec{\sigma}}}}_{0{\varvec{e}}{\varvec{k}}}{e}^{-\frac{t}{{\tau }_{Rck}}}+{{\widetilde{{\varvec{\sigma}}}}_{{\varvec{r}}{\varvec{e}}{\varvec{k}}}}^{CCM}\left(r,\tau \right)\left(1-{e}^{-\frac{t}{{\tau }_{Rck}}}\right)$ Cell residual stress is purely dissipative. ${{{\widetilde{{\varvec{\sigma}}}}_{{\varvec{e}}{\varvec{k}}}}^{CCM}=\eta }_{{\varvec{c}}{\varvec{k}}}{\dot{\widetilde{{\varvec{\varepsilon}}}}}_{{\varvec{e}}{\varvec{k}}}$

where $k\equiv S,V$, $S$ is shear, $V$ is volumetric, ${\tau }_{Rck}$ is the cell stress relaxation time, ${E}_{ck}$ is the elastic modulus, ${\upeta }_{ck}$ is the cell viscosity (shear or bulk), $r$ is the space coordinate, $t$ is a short-time scale (i.e. minutes), $\tau$ is a long-time-scale (i.e. hours), $\Vert {\overrightarrow{{\varvec{v}}}}_{{\varvec{e}}}\Vert$ is the cell speed, ${\overrightarrow{{\varvec{v}}}}_{{\varvec{e}}}$ is the cell velocity equal to ${\overrightarrow{{\varvec{v}}}}_{{\varvec{e}}}=\frac{{\varvec{d}}\overrightarrow{{\varvec{u}}}}{{\varvec{d}}{\varvec{\tau}}}$**,** $\overrightarrow{{\varvec{u}}}\left(r,\tau \right)$ is the cell local displacement field, ${{\widetilde{{\varvec{\sigma}}}}_{{\varvec{e}}{\varvec{k}}}}^{CCM}\left(r,t,\tau \right)$ is the cell stress (normal or shear), ${{\dot{\widetilde{{\varvec{\sigma}}}}}_{{\varvec{e}}{\varvec{k}}}}^{CCM}$ is the rate of stress change ${{\dot{\widetilde{{\varvec{\sigma}}}}}_{{\varvec{e}}{\varvec{k}}}}^{CCM}=\frac{d{{\widetilde{{\varvec{\sigma}}}}_{{\varvec{e}}{\varvec{k}}}}^{CCM}}{dt}$ caused by the stress relaxation, ${\widetilde{{\varvec{\varepsilon}}}}_{{\varvec{c}}{\varvec{k}}}$ is the cell strain such that the volumetric strain is equal to ${\widetilde{{\varvec{\varepsilon}}}}_{{\varvec{e}}{\varvec{V}}}\left(r,\tau \right)=\overrightarrow{(\nabla }\cdot \overrightarrow{{\varvec{u}}})\widetilde{{\varvec{I}}}$**,** $\widetilde{{\varvec{I}}}$ is the unit tensor, and the shear strain ${\widetilde{{\varvec{\varepsilon}}}}_{{\varvec{e}}{\varvec{S}}}\left(r,\tau \right)=\frac{1}{2}\left(\overrightarrow{\nabla }\overrightarrow{{\varvec{u}}}+{\overrightarrow{\nabla }\overrightarrow{{\varvec{u}}}}^{{\varvec{T}}}\right)$**,** ${\dot{\widetilde{{\varvec{\varepsilon}}}}}_{{\varvec{c}}{\varvec{k}}}$ is the corresponding strain rate equal to ${\dot{\widetilde{{\varvec{\varepsilon}}}}}_{{\varvec{e}}{\varvec{k}}}=\frac{d{\widetilde{{\varvec{\varepsilon}}}}_{{\varvec{e}}{\varvec{k}}}}{d\tau }$, ${\upeta }_{\alpha k}$ is the effective modulus, ${D}^{\alpha } \widetilde{{\varvec{\varepsilon}}}\left(r,\tau \right)=\frac{{d}^{\alpha }\widetilde{{\varvec{\varepsilon}}}\left(r,\tau \right)}{d{\tau }^{\alpha }}$ is the fractional derivative, and $\mathrm{\alpha }$ gives the order of fractional derivatives (the damping coefficient). Caputo’s definition of the fractional derivative of a function $\widetilde{{\varvec{\varepsilon}}}\left(r,\tau \right)$ is used and expressed as: ${D}^{\alpha }\widetilde{{\varvec{\varepsilon}}}=\frac{1}{\Gamma\left(1-\alpha \right)}\frac{d}{dt}{\int }_{0}^{t}\frac{\widetilde{{\varvec{\varepsilon}}}\left({r,\tau }{\prime}\right)}{{\left(\tau -\tau {\prime}\right)}^{\alpha }}d\tau {\prime}$ (where Г $\left(1-\alpha \right)$ is a gamma function) [77]

An increase in the cell packing density, caused by a collision of velocity fronts, results in an increase in the cell compressive stress and changes the mechanism of cell migration from the convective through diffusive to sub-diffusive which corresponds to the state of a multicellular system near jamming. The viscoelasticity of epithelial systems corresponds to linear constitutive models, i.e. the Zener model for the convective regime and the Kelvin-Voigt model for the conductive regime. The system near jamming represents a particularly-defined nonlinear viscoelastic solid state which satisfies following conditions: (1) the viscosity increases dramatically, (2) the stress relaxation time tends to infinity, and (3) the ratio between the storage and loss moduli $G{\prime}$ and $G{\prime}{\prime}$, respectively is constant and higher than unity. Here, $G{\prime}$ represents a measure of the stored elastic energy within the system, while $G{\prime}{\prime}$ represents a a measure of energy dissipation caused by the cell rearrangement [28, 80].

An increase in the cell packing density leads to the stiffening of multicellular systems if and only if cells retain their contractile state and strength of cell–cell adhesion contacts [51]. However, when cells jam, they enter the passive non-contractile state accompanied by a weakening of cell–cell adhesion contacts, resulting in energy dissipation and, consequently, system softening [51]. After energy dissipation, cells re-establish strong cell–cell adhesion contacts and start migrating again, leading to an increase in the cell stiffness [51]. This local stiffening and softening is capable of destabilising the flow and inducing long-time inertial effects.

The epithelial-to-mesenchymal transition also induces local softening of multicellular system. It is in accordance with the fact that the mesenchymal cells establish weak N-cadherin-mediated cell–cell adhesion contacts, while the epithelial cells establish strong E-cadherin-mediated cell–cell adhesion contacts [17]. Accordingly, with the reversibility of epithelial-to-mesenchymal transition, the mesenchymal cells can reach out the epithelial phenotype again leading to stiffening of a multicellular system [17].

Consequently, a change in the state of viscoelasticity, caused by the generation of compressive stress, is the one of the main origins of the low-${R}_{e}$ turbulence in the form of the propagating waves, while the epithelial-to-mesenchymal transition can induce the generation of standing waves. As discussed above, the generation of standing waves is related to cell swirling motion [7, 9]. It occurs in two inter-connected steps: (1) the epithelial-to-mesenchymal cell state transition results in a weakening of the cell–cell adhesion contacts and a decrease in the cell cohesiveness, while cells retain their active, contractile state, and (2) the shear stress torque $\Delta \overrightarrow{{\varvec{T}}}$ does work ${{\varvec{W}}}_{{\varvec{e}}}=\Delta \overrightarrow{{\varvec{T}}}\cdot {\overrightarrow{{\varvec{\omega}}}}_{{\varvec{e}}}$ (where ${\overrightarrow{{\varvec{\omega}}}}_{{\varvec{e}}}=\overrightarrow{\nabla }X{\overrightarrow{{\varvec{v}}}}_{{\varvec{e}}}$ is the angular velocity) and induces the swirling motion of less cohesive cellular systems [9]. The lifetime of migrated cell clusters within the cell monolayers can be identified with the period of long-time oscillations which is approximately equal to $4-6 {\text{hours}}$ [6, 7].

We will discuss the mechanism of generation of long-time inertial effects during collective cell migration, based on conservation of cell momentum and mass equations, by considering our two model systems: (1) the fusion of two cell aggregates, and (2) free expansion of an epithelial monolayer on a surface matrix.

### Generation of long-time inertial effects during the fusion of two cell aggregates

**Fig. 5:**
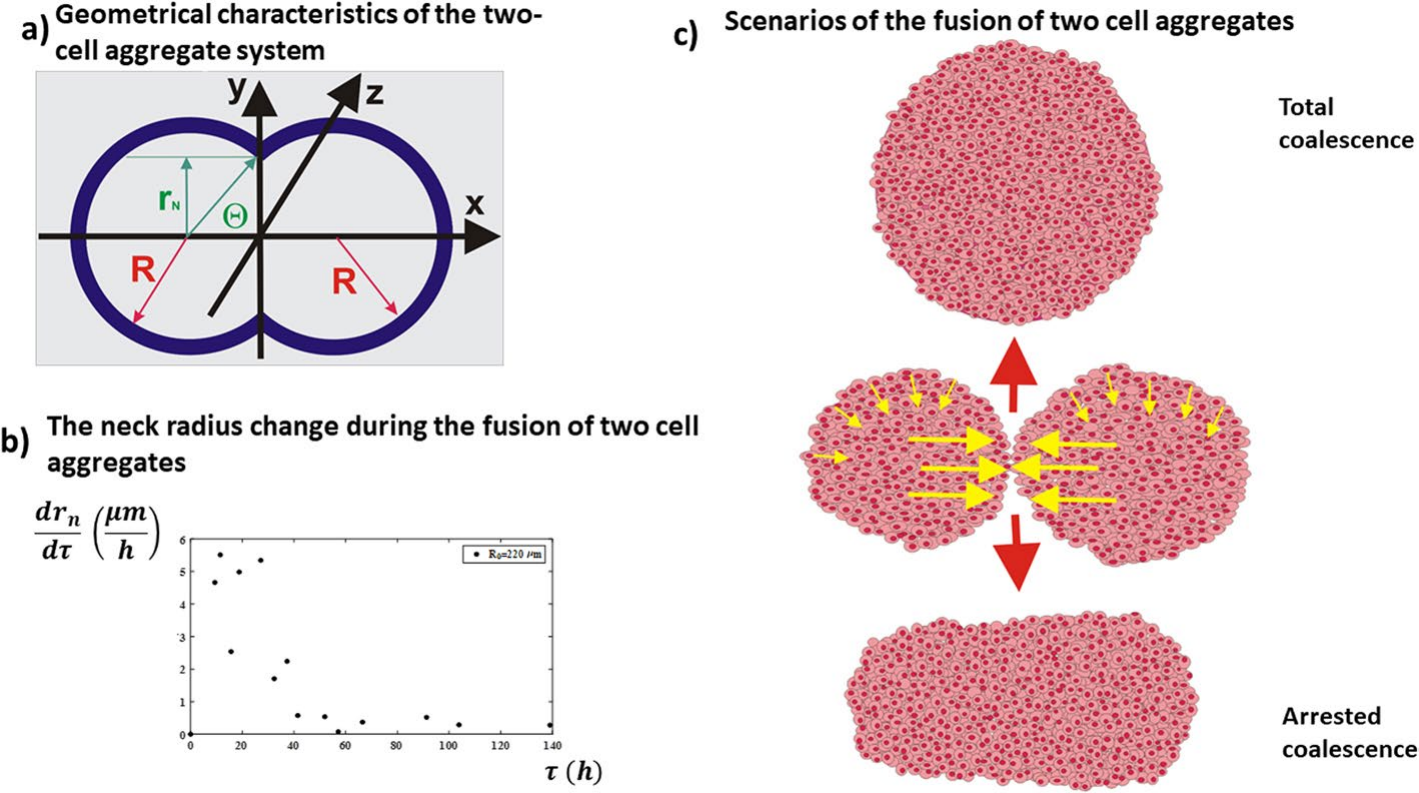
The fusion of two cell aggregates: (**a**) geometry of the two-aggregate systems, (**b**) oscillatory decrease the rate of the neck radius change (the rate of the neck radius change was calculated from the experiments by Shafiee et al. [81]), (**c**) two possible scenarios for the fusion of a pair of cell aggregates: total coalescence and arrested coalescence

The fusion of two epithelial aggregates is driven by the epithelial surface tension and it leads to a decrease in the surface and volume of the two-aggregate system. The epithelial surface tension causes cell migration from the aggregates’ surfaces toward the core regions and oppositely-directed flows from the core regions of the aggregates toward their mutual contact point as shown in Fig. 5.

The collision of the velocity fronts causes an increase in the cell compressive stress at the contact point between the cell aggregates, resulting in a sequence of local jamming-to-unjamming transitions caused by an oscillatory variation in the cell velocity [21, 22]. In addition, the cell velocity, cell packing density, and neck radius also perform long-time oscillations [27]. The neck in this case represents the contact region between two aggregates. The long-time oscillations of cell velocity area consequence of the competition between the driving and resistive forces.

The driving force for the fusion of two cell aggregates is the surface tension, formulated by Pajic-Lijakovic and Milivojevic [22] as: ${n}_{e}{{\overrightarrow{{\varvec{F}}}}_{{\varvec{s}}{\varvec{t}}}}^{{\varvec{e}}}={n}_{e}{\gamma }_{e}\overrightarrow{{\varvec{u}}}$ (where $\overrightarrow{{\varvec{u}}}$ is the cell displacement field caused by collective cell migration). The surface tension force guides cell movement from the aggregates’ surface regions toward their core regions and guides the migration of cells from the core regions toward the contact area between two aggregates in order to decrease the surface of two-aggregate system. The resistive force is the viscoelastic force ${{\overrightarrow{{\varvec{F}}}}_{{\varvec{T}}{\varvec{v}}{\varvec{e}}}}^{{\varvec{e}}}$ based on a modified form of the model due to Murray et al. [76] as: ${{\overrightarrow{{\varvec{F}}}}_{{\varvec{T}}{\varvec{v}}{\varvec{e}}}}^{{\varvec{e}}}=\nabla \cdot \left({\widetilde{{\varvec{\sigma}}}}_{{\varvec{e}}{\varvec{r}}\boldsymbol{\varvec{T}}}+{{\widetilde{{\varvec{\sigma}}}}_{\boldsymbol{\varvec{e}}}}^{SD}\right)$, where ${\widetilde{{\varvec{\sigma}}}}_{{\varvec{e}}{\varvec{r}}\boldsymbol{\varvec{T}}}$ is the total cell residual stress caused by surface effects and collective cell migration, while ${{\widetilde{{\varvec{\sigma}}}}_{\boldsymbol{\varvec{e}}}}^{SD}$ is the solid stress accumulated within the core regions of the aggregates. The viscoelastic force is a measure of the accumulation of the elastic energy, rather than energy dissipation. It is known that the cell residual stress, caused by collective cell migration, is purely elastic for a cell packing density ${n}_{e}\le {n}_{conf}$ (Table 1). The solid stress results from cell growth under confluent condition in the cell aggregate core region [82]. The accumulation of elastic energy reduces the movement of epithelial cells [70, 71].

The conservation of momentum equation can be formulated as [23]:

9 $${\langle m\rangle }_{e}\frac{D\left[{n}_{e}\left(r,\tau \right){\overrightarrow{{\varvec{v}}}}_{{\varvec{e}}}\left(r,\tau \right)\right]}{D\tau }={n}_{e}{{\overrightarrow{{\varvec{F}}}}_{{\varvec{s}}{\varvec{t}}}}^{{\varvec{e}}}-{{\overrightarrow{{\varvec{F}}}}_{{\varvec{T}}{\varvec{v}}{\varvec{e}}}}^{{\varvec{e}}}$$

where $\tau$ is the long-time,${\langle m\rangle }_{e}$ is the average mass of a single epithelial cell, the epithelial cell velocity ${\overrightarrow{{\varvec{v}}}}_{{\varvec{e}}}\left(r,\tau \right)=\frac{d\overrightarrow{{\varvec{u}}}}{d\tau }$, $\overrightarrow{{\varvec{u}}}$ is the epithelial cell displacement field, and the material derivative $\frac{D\left[{n}_{e}\left(r,\tau \right){\overrightarrow{{\varvec{v}}}}_{{\varvec{e}}}\left(r,\tau \right)\right]}{D\tau }={n}_{e}\left[\frac{\partial {\overrightarrow{{\varvec{v}}}}_{{\varvec{e}}}}{\partial \tau }+({\overrightarrow{{\varvec{v}}}}_{{\varvec{e}}}\cdot \overrightarrow{\nabla }){\overrightarrow{{\varvec{v}}}}_{{\varvec{e}}}\right]+{\overrightarrow{{\varvec{v}}}}_{{\varvec{e}}}\left[\frac{\partial {n}_{e}}{\partial \tau }+({\overrightarrow{{\varvec{v}}}}_{{\varvec{e}}}\cdot \overrightarrow{\nabla }){n}_{e}\right]$ [1]. Multicellular systems are compressible. Shafiee et al. [81] considered the fusion of two confluent skin fibroblast cell aggregates and pointed out that the surface of the two-aggregate systems decreases by a factor of 2.18 × , while their volume decreases by 2.38 × within 140 h. A cancer cell spheroid of CT26 cells lost 15% of its volume under an osmotic stress of 5 kPa, while the cell volumes were approximately constant [83]. This stress corresponds to that under physiological conditions. However, cells have special mechanisms to regulate the cell packing density such as: the contact inhibition of locomotion, cell extrusion, and remodeling of cell–cell adhesion contacts [17, 50, 84]. A small change in the cell packing density induces significant change in the cell velocity. Cell speed can be correlated with cell packing density in the form of $\Vert {\overrightarrow{{\varvec{v}}}}_{{\varvec{e}}}\Vert \sim {{n}_{e}}^{-b}$ (where $b$ is the scaling exponent). The scaling exponent is: (1) $b=1.85$ for free expansion of MDCK cells [55] and (2) $b=2.35$ for free expansion of MCF-10A cells [54]. Consequently, we can suppose that${n}_{e}\left[\frac{\partial {\overrightarrow{{\varvec{v}}}}_{{\varvec{e}}}}{\partial \tau }+({\overrightarrow{{\varvec{v}}}}_{{\varvec{e}}}\cdot \overrightarrow{\nabla }){\overrightarrow{{\varvec{v}}}}_{{\varvec{e}}}\right]\gg {\overrightarrow{{\varvec{v}}}}_{{\varvec{e}}}\left[\frac{\partial {n}_{e}}{\partial \tau }+({\overrightarrow{{\varvec{v}}}}_{{\varvec{e}}}\cdot \overrightarrow{\nabla }){n}_{e}\right]$**.**

The long time inertial effects appear when the dynamical equilibrium is perturbed, i.e. ${n}_{e}{{\overrightarrow{{\varvec{F}}}}_{{\varvec{s}}{\varvec{t}}}}^{{\varvec{e}}}\ne {{\overrightarrow{{\varvec{F}}}}_{{\varvec{T}}{\varvec{v}}{\varvec{e}}}}^{{\varvec{e}}}$. The mechanism of the long-time oscillations in cell velocity involves a sequence of inter-connected steps: (1) the surface tension acts to decrease the surface and volume of the two-aggregate system by inducing an increase in the cell compressive stress, (2) the accumulation of cell compressive stress causes a decrease in the cell velocity and an increase in the cell packing density, (3) the increase in the cell packing density intensifies cell–cell interactions, inhibiting locomotion by direct contact (4) these interactions result in a weakening of cell–cell adhesion contacts, (5) this weakening of cell–cell adhesion contacts causes energy dissipation, leading to a decrease in the cell compressive stress, and (6) the cells re-establish strong E-cadherin-mediated cell–cell adhesion contacts and start migration again by increasing their velocity.

In addition to the conservation of momentum equation, we also require conservation of mass equation for the movement of epithelial cells within the clusters, which can be expressed as:

10 $$\frac{\partial {n}_{e}(r,\tau )}{\partial \tau }=\overrightarrow{\nabla }{\cdot {\overrightarrow{({\varvec{J}}}}_{{\varvec{m}}}}^{{\varvec{e}}}+{\overrightarrow{{\varvec{J}}}}_{{\varvec{M}}{\varvec{e}}})$$

where the flux ${{\overrightarrow{{\varvec{J}}}}_{{\varvec{m}}}}^{{\varvec{e}}}$ describes the mode of cell movement. For the convective movement of cells it is the convective flux ${{\overrightarrow{{\varvec{J}}}}_{{\varvec{m}}}}^{{\varvec{e}}}\equiv {{\overrightarrow{{\varvec{J}}}}_{{\varvec{c}}{\varvec{o}}{\varvec{n}}{\varvec{v}}}}^{{\varvec{e}}}={n}_{e}{\overrightarrow{{\varvec{v}}}}_{{\varvec{e}}}$**.** For the diffusion mechanism of cell movement under higher cell packing density it is the diffusion flux ${{\overrightarrow{{\varvec{J}}}}_{{\varvec{m}}}}^{{\varvec{e}}}\equiv {{\overrightarrow{{\varvec{J}}}}_{{\varvec{d}}{\varvec{i}}{\varvec{f}}{\varvec{f}}}}^{{\varvec{e}}}$ $=-{D}_{eff}\overrightarrow{\nabla }{n}_{e}$, where ${D}_{eff}$ is the effective diffusion coefficient. For high cell packing density near to cell-jamming, the cell migration is damped and corresponds to sub-diffusion. In this case, the corresponding flux ${{\overrightarrow{{\varvec{J}}}}_{{\varvec{m}}}}^{{\varvec{e}}}$ represents the sub-diffusion flux ${{\overrightarrow{{\varvec{J}}}}_{{\varvec{m}}}}^{{\varvec{e}}}\equiv {{\overrightarrow{{\varvec{J}}}}_{{\varvec{s}}{\varvec{u}}{\varvec{b}}-{\varvec{d}}{\varvec{i}}{\varvec{f}}{\varvec{f}}}}^{{\varvec{e}}}$ and the conservation of mass equation (Eq. 9) should be transformed to ${D}^{\alpha }{n}_{e}=\overrightarrow{\nabla }{\cdot {\overrightarrow{({\varvec{J}}}}_{{\varvec{d}}\boldsymbol{\varvec{s}}{\varvec{u}}{\varvec{b}}-{\varvec{d}}{\varvec{i}}{\varvec{f}}{\varvec{f}}}}^{{\varvec{e}}}+{\overrightarrow{{\varvec{J}}}}_{{\varvec{M}}{\varvec{e}}})$, where ${D}^{\alpha }{n}_{e}$ is the fractional derivative expressed as ${D}^{\alpha }\left({n}_{e}\right)=\frac{{d}^{\alpha }{n}_{e}}{d{\tau }^{\alpha }}$, and $\mathrm{\alpha }$ is the order of the fractional derivative *i.e.* the damping coefficient of the system structural changes which satisfies the condition$\alpha \le 1/2$, the flux${{\overrightarrow{{\varvec{J}}}}_{{\varvec{d}}\boldsymbol{\varvec{s}}{\varvec{u}}{\varvec{b}}-{\varvec{d}}{\varvec{i}}{\varvec{f}}{\varvec{f}}}}^{{\varvec{e}}}=-{D}_{\alpha }\overrightarrow{\nabla }{n}_{e}$, and ${D}_{\alpha }$ is the the damped-conductive diffusion coefficient which has units of $\frac{{{\text{m}}}^{2}}{{{\text{s}}}^{\mathrm{\alpha }}}$ [28].

The flux ${\overrightarrow{{\varvec{J}}}}_{{\varvec{M}}{\varvec{e}}}$ is the Marangoni flux which depends on the gradient of the epithelial surface tension and has been expressed as: ${\overrightarrow{{\varvec{J}}}}_{{\varvec{M}}{\varvec{e}}}={k}_{Me}{n}_{e}{\overrightarrow{\nabla }}_{{\varvec{s}}}{\gamma }_{e}$, where ${k}_{Me}$ is the measure of the mobility of epithelial cells along the biointerface and ${\overrightarrow{\nabla }}_{{\varvec{s}}}\left(\cdot \right)$ is the surface gradient [62]. The Marangoni flux directs the movement of cells from the region of lower interfacial tension toward the region of larger interfacial tension. The Marangoni flux also arises in a variety of soft matter systems, primarily as a consequence of the temperature distribution [78].

The low-${R}_{e}$ turbulence (i.e.${R}_{e}\ll 1$), in this case, could be characterized by modified Weissenberg number ${W}_{i app}={T}_{c}{(\frac{{r}_{N/{r}_{Neq}}}{d\tau })}_{max}$, where ${T}_{c}$ is the period of the long-time oscillations in the cell velocity, ${r}_{N}$ is the neck radius, and ${r}_{Neq}$ is the equilibrium neck radius obtained when the fusion is finished or arrested. The neck radius can be expressed as: ${r}_{N}\left(\tau \right)=R\left(\tau \right){\text{sin}}\theta \left(\tau \right)$, where $R\left(\tau \right)$ is the aggregate radius and $\theta \left(\tau \right)$ is the fusion angle which changes from $\theta \left(0\right)=0$ to $\theta \left(\infty \right)=\frac{\pi }{2}$ as shown in Fig. 3 [85, 86].The Weber number, which represents the ratio between kinetic energy and surface energy, is formulated here in this case as: ${W}_{e}=\frac{{\langle m\rangle }_{e}\langle {n}_{e eq}\rangle {\Vert {\overrightarrow{{\varvec{v}}}}_{{\varvec{e}}{\varvec{M}}{\varvec{A}}{\varvec{X}}}\Vert }^{2}}{{\gamma }_{e eq}\Delta A}$, where ${\gamma }_{e eq}$ is the equilibrium epithelial surface tension obtained after the fusion of two cell aggregate, $\Delta A$ is the decrease in the surface area of the two-aggregate system, the average cell packing density at the end of the fusion $\langle {n}_{e eq}\rangle =\frac{1}{{V}_{eq}}\int {n}_{e}\left(r,{\tau }_{eq}\right)d{r}^{3},$ ${\tau }_{eq}$ is the equilibrium time for fusion, and $\Vert {\overrightarrow{{\varvec{v}}}}_{{\varvec{e}}{\varvec{M}}{\varvec{A}}{\varvec{X}}}\Vert$ is the maximum cell speed.

To complement our discussion of the fusion of two epithelial aggregates, we now consider the generation of long-time inertial effects on another model system: the free expansion of epithelial monolayers on a substrate matrix.

### Generation of long-time inertial effects during free expansion of epithelial monolayers

Free expansion of epithelial monolayers on a substrate matrix can be treated as an interfacial problem. For characterizing the cell rearrangement caused by the collective cell migration, it is necessary to account for the viscoelasticity and surface characteristics of the multicellular system and matrix based on the inter-relation between the physical parameters such as: the epithelial and matrix surface tensions, the epithelial-matrix interfacial tension, the cell residual stress and the matrix residual stress. The long-time oscillations in cell velocity are connected with the monolayer oscillatory wetting and they arise from a competition between the driving forces and resistive forces. The driving forces are the interfacial tension and the mixing force, whereas the viscoelastic force and traction force constitute the resistive forces.

The mixing force ${{\overrightarrow{{\varvec{F}}}}_{{\varvec{m}}{\varvec{i}}{\varvec{x}}}}^{{\varvec{e}}-{\varvec{m}}}$ results from the thermodynamic energetic effect of mixing of two soft matter systems, in this case the cell monolayer and the substrate matrix. This force may be formulated as: ${{\overrightarrow{{\varvec{F}}}}_{{\varvec{m}}{\varvec{i}}{\varvec{x}}}}^{{\varvec{c}}-{\varvec{m}}}=\frac{1}{{h}_{c}}{\overrightarrow{\nabla }}_{s}\left({e}_{a}\right)$, where ${h}_{c}$ is the average size of single cell. The interfacial tension force ${n}_{e}{{\overrightarrow{{\varvec{F}}}}_{{\varvec{i}}{\varvec{t}}}}^{{\varvec{e}}-{\varvec{m}}}$ drives cell wetting, depending on the inter-relation between the tissue and matrix surface tensions accompanied by the interfacial tension between them, expressed in the form of the cell spreading factor [72]. This force is expressed as: ${n}_{e}{{\overrightarrow{{\varvec{F}}}}_{{\varvec{i}}{\varvec{t}}}}^{{\varvec{e}}-{\varvec{m}}}={n}_{e}{S}^{e}\overrightarrow{{\varvec{u}}}$, where the spreading factor of epithelial cells ${S}^{e}={\gamma }_{m}-\left({\gamma }_{e}+{\gamma }_{em}\right)$ and $\overrightarrow{{\varvec{u}}}$ is the cell displacement field. The spreading factor for free expansion of cell monolayers satisfies the condition that ${S}^{e}>0$ and change with time causes an oscillatory wetting [6].

The traction force is the resistive force formulated by Murray et al. [76] as: ${\rho }_{e-m}{{\overrightarrow{{\varvec{F}}}}_{{\varvec{t}}{\varvec{r}}}}^{{\varvec{e}}-{\varvec{m}}}={\rho }_{e-m}{k}_{c}{\overrightarrow{{\varvec{u}}}}_{{\varvec{m}}}$ (where ${\rho }_{e-m}$ is the number density of cell–matrix adhesion contacts, ${k}_{c}$ is the spring constant of single cell–matrix adhesion contact, and ${\overrightarrow{{\varvec{u}}}}_{{\varvec{m}}}$ is the local displacement of matrix caused by cell tractions). It is in accordance with fact that establishment of strong cell–matrix adhesion contacts can reduce cell movement [17]. The viscoelastic force as formulated by Murray et al. [76] may be expressed as: ${{\overrightarrow{{\varvec{F}}}}_{{\varvec{v}}{\varvec{e}}}}^{{\varvec{e}}}=\nabla \cdot {(\widetilde{{\varvec{\sigma}}}}_{{\varvec{e}}{\varvec{r}}{\varvec{T}}}-{\widetilde{{\varvec{\sigma}}}}_{{\varvec{m}}{\varvec{r}}})$ (where ${\widetilde{{\varvec{\sigma}}}}_{{\varvec{e}}{\varvec{r}}{\varvec{T}}}$ is the total cell residual stress and ${\widetilde{{\varvec{\sigma}}}}_{{\varvec{m}}{\varvec{r}}}$ is the residual stress within a substrate matrix caused by cell tractions). The residual stress within a substrate matrix depends on the matrix viscoelasticity and cell tractions. Cell tractions also influence the matrix surface tension and epithelial-matrix interfacial tension.

The conservation of momentum equation can be expressed as:

11 $${{\langle m\rangle }_{e}n}_{e}\left(\boldsymbol{r},\tau \right)\frac{D{\overrightarrow{{\varvec{v}}}}_{{\varvec{e}}}\left(\boldsymbol{r},\tau \right)}{D\tau }={{\overrightarrow{{\varvec{F}}}}_{{\varvec{m}}}}^{{\varvec{e}}}+{n}_{e}{{\overrightarrow{{\varvec{F}}}}_{{\varvec{i}}{\varvec{t}}}}^{{\varvec{e}}}-{{\overrightarrow{{\varvec{F}}}}_{{\varvec{T}}{\varvec{v}}{\varvec{e}}}}^{{\varvec{e}}-{\varvec{m}}}-{\rho }_{e-m}{{\overrightarrow{{\varvec{F}}}}_{{\varvec{t}}{\varvec{r}}}}^{{\varvec{e}}-{\varvec{m}}}$$

where $\frac{D{\overrightarrow{{\varvec{v}}}}_{{\varvec{e}}}}{D\tau }=\frac{\partial {\overrightarrow{{\varvec{v}}}}_{{\varvec{e}}}}{\partial \tau }+({\overrightarrow{{\varvec{v}}}}_{{\varvec{e}}}\cdot \overrightarrow{\nabla }){\overrightarrow{{\varvec{v}}}}_{{\varvec{e}}}$ is the material derivative [1]. In this case, the long-time inertial effects appear when the dynamical equilibrium between the driving and resistive forces is perturbed. The mechanism of the long-time oscillations in the cell velocity includes several inter-connected steps: (1) the epithelial monolayer undergoes wetting for the case that ${S}^{e}>0$, (2) altered extension cause an increase in the cell tensional residual stress and epithelial surface tension which result in a decrease in the cell velocity, (3) local increase in the epithelial surface tension leads to a decrease in the spreading coefficient and slows down the wetting, (4) the wetting slow-down results in a decrease in the tensional stress accompanied by the epithelial surface tension, (5) a decrease in the surface tension causes an increase in the spreading factor and cell velocity again. The cell dynamics described can be treated as a damped oscillatory wetting up to the equilibrium state. The cell tensional stress performs long-time oscillations. The maximum tensional stress was $\sim 300 {\text{Pa}}$ [6].

**Fig. 6:**
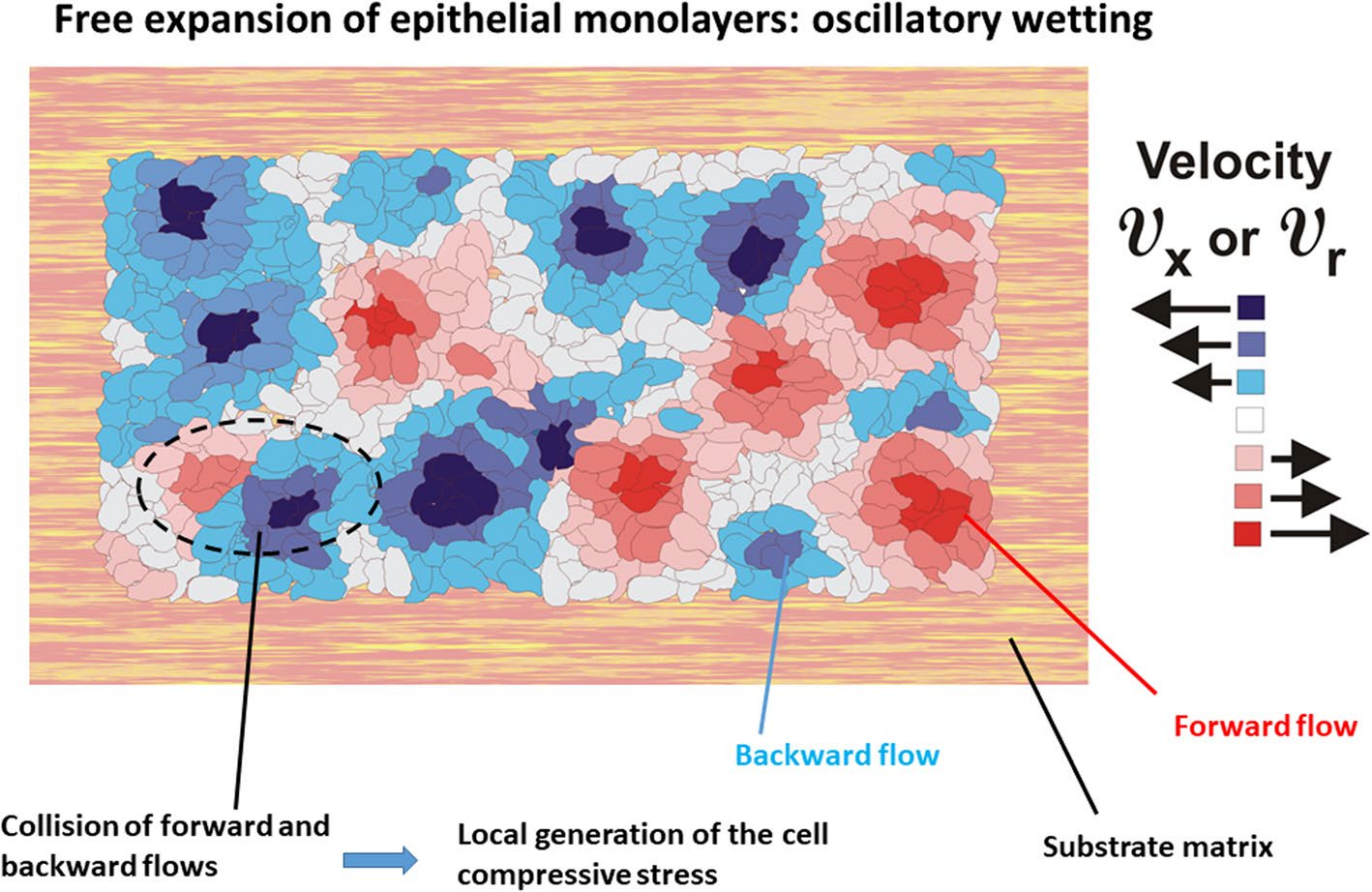
Schematic presentation of the generation of local forward and backward flows during free expansion of epithelial monolayers on a substrate matrix, inspired by the experimental data of Serra-Picamal et al. [6]

An inhomogeneous distribution of cell residual stress accompanied by the epithelial surface tension causes inhomogeneous wetting of epithelial monolayers, inducing the generation of local forward and backward flows as shown in Fig. 6:

The generation of forward and backward flows was experimentally confirmed by Serra-Picamal et al. [6]. These flows could represent a local de-wetting of the monolayers. The collisions between forward and backward flows can induce an increase in the cell compressive stress and even lead to the local cell jamming-state transition. However, the magnitude of the compressive stress generated during fusion of cell aggregates should be much larger and capable of causing the arrested coalescence, which represents the global cell jamming-state transition within the contact region between two cell aggregates [21]. The distribution of kinetic energy within migrating epithelial monolayers corresponds to a q-Gaussian distribution with $q\approx 1.2$ [10]. This type of distribution points to the presence of multiplicative noise, perhaps caused by the forward and backward cell flow.

The conservation of mass equation in the case of free extension of epithelial monolayers could be also described by Eq. 9, while the Marangoni flux in this case is induced by the gradient of epithelial-matrix interfacial tension and can be expressed as: ${\overrightarrow{{\varvec{J}}}}_{{\varvec{M}}{\varvec{e}}}={k}_{Me}{n}_{e}{\overrightarrow{\nabla }}_{{\varvec{s}}}{\gamma }_{em}$.

The low-${R}_{e}$ turbulence (i.e.${R}_{e}\ll 1$), in this case, could be characterized by modified Weissenberg number ${W}_{i app}$ expressed as: ${W}_{i app}={T}_{c}{(\frac{{\Delta L}_{/{L}_{0}}}{d\tau })}_{max}$, where ${T}_{c}$ is the period of long-time oscillations of cell velocity,$\frac{\Delta L}{{L}_{0}}$ is the change of the monolayer width relative to the initial width${L}_{0}$. The Weber number could be formulated as: ${W}_{e}=\frac{{\langle m\rangle }_{e}\langle {n}_{e eq}\rangle {\Vert {\overrightarrow{{\varvec{v}}}}_{{\varvec{e}}{\varvec{M}}{\varvec{A}}{\varvec{X}}}\Vert }^{2}}{{\gamma }_{em eq}\Delta A}$, where ${\gamma }_{em eq}$ is the equilibrium epithelial-matrix interfacial tension and $\Delta A$ is the extension of the cell monolayer area during the process of the oscillatory wetting.

Consequently, the long-time inertial effects in this case are induced by oscillatory changes in the epithelial surface tension and the cell residual stress accumulation.

## Comparative analysis of three type of systems from the standpoint of turbulence

Three types of systems have been discussed above in the context of turbulence: (1) high-${R}_{e}$ turbulence of Newtonian fluids, (2) low- and moderate-${R}_{e}$ turbulence of polymer solutions, and (3) low-${R}_{e}$ turbulence caused by the migration of epithelial collectives. The generation of flow instabilities, a hallmark of turbulence, is primarily connected with the system’s rheological behaviour. A summary of the main characteristics of turbulence for various systems is given in Table 2:

**Table 2:** The main characteristics of turbulence for various systems

	Newtonian fluids	Polymer solutions	Epithelial multicellular systems
compressibility	incompressible	incompressible	compressible
Rheological behaviour	Turbulent liquids	Viscoelastic liquids	-Viscoelastic solids for epithelial phenotype -Viscoelastic liquid for mesenchymal phenotype
Linearity of the constitutive model	Nonlinear	Nonlinear	-Linear for cell packing density ${n}_{e}<{n}_{j}$ -Non-linear for the cell state near jamming, i.e. when ${n}_{e}\to {n}_{j}$ (where ${n}_{j}$ is the cell packing density in the jamming state)
Main characteristics of the constitutive models	Stress and strain cannot relax Changes of stress and strain occur on the same time-scale	Stress can relax under constant strain condition Strain cannot relax Changes of stress and strain occur on the same time-scale	Stress can relax for the condition that ${n}_{e}={n}_{conf}$ In this case stress relaxes under: (1) constant strain rate conditions for viscoelastic liquids and (2) constant strain for viscoelastic solids The stress relaxation occurs on a time scale of minutes, while strain change and residual stress generation occur on a time scale of hours
Reynolds number	For Couette flow ${R}_{eo}=0$ ${R}_{ei}>1500$	For Couette flow ${R}_{eo}=0$ ${R}_{e}<1$ for elastic turbulence ${R}_{ei}\ge 200$ for elasto-inertial turbulence	${R}_{e}\ll 1$
Inertial effects	Short-time inertial effects	The elastic turbulence is inertia-less unstable flow of polymer solutions The elasto-inertial turbulence is a product of inertial effects	Long-time inertial effects (the effective inertia)

where ${n}_{e}$ is the cell packing density, ${n}_{conf}$ is the cell packing density at the confluent state, ${n}_{j}$ is the cell packing density at the jamming state, ${R}_{eo}$ and ${R}_{ei}$ are Reynolds numbers of the outer and inner cylinder, respectively

Turbulence is associated with the nonlinear behaviour of an out-of-equilibrium physical system whose energy is distributed over a large number of degrees of freedom. The nonlinearity is caused by inertial effects and can also be induced by nonlinear rheological behaviour. Inertial effects lead to nonlinearity, which is associated with the second term of the material derivative, i.e. $(\overrightarrow{{\varvec{v}}}\cdot \overrightarrow{\nabla })\overrightarrow{{\varvec{v}}}$**.** The rheological behavior of Newtonian fluids under high-${R}_{e}$ number and polymer solutions under low and moderate-${R}_{e}$ number is nonlinear. The nonlinear rheological behaviour of Newtonian fluids under high-${R}_{e}$ number is primarily induced by velocity fluctuations. In the case of polymer solutions, the nonlinear behaviour is induced by polymer stretching, which results in the generation of tensional ‘’hoop’’ stress and chain stiffening. However, the generation of flow instabilities during collective cell migration is related to the density-driven changes of the state of viscoelasticity. The inhomogeneous distribution of cell packing density, accompanied by cell mechanical stress, tissue stiffness, and epithelial surface tension have a feedback impact on the cell velocity. The distribution of cell velocity within migrating epithelial collectives is significantly inhomogeneous. When cell migration aligns with the sub-diffusion mechanism close to cell jamming, the rheological behaviour of epithelial systems transitions from linear to nonlinear. Cells actively change the magnitude of accumulated stress by remodelling cell–cell adhesion contacts and changing the state of contractility.

## Conclusion

This theoretical review has clarified the role of viscoelasticity in the appearance of low-Reynolds turbulence. Three types of system were considered and compared: (1) high-Reynolds turbulent flow of Newtonian fluids; (2) low-Reynolds flow of polymer solutions; and (3) migration of epithelial collectives, which also represents an example of low-Reynolds turbulence. The main results were obtained by integrating physical models with experiments on fluid mechanics, bio-mechanics, and biological physics. We can summarize them as follows:

- Inertial effects, as a hallmark of turbulent flow, appear as the consequence of a perturbation of the dynamical equilibrium induced by an imbalance between driving forces and resistive forces. While the imbalance is induced by an inhomogeneous distribution of kinetic energy for the case of Newtonian fluids, it is caused by an inhomogeneous distribution of stored elastic energy and energy dissipation for the case of viscoelastic systems such as: polymer solutions and cellular systems.
- The driving force for the shear flow of Newtonian fluids is frequently induced externally by a pressure gradient, while the resistive, viscous force depends on the viscosity of eddies and the geometry of the flow. Intensive fluctuations in the viscosity of eddies, accompanied by energy dissipation, are the main generators of inertial effects.
- While the imbalance between driving and resistive forces occurs at high Reynolds numbers for Newtonian fluids, this imbalance can be induced at moderate Reynolds number during flow of polymer solutions as a consequence of their viscoelasticity. Shear flow of viscoelastic liquids, such as polymer solutions, generates extensional strain rate perpendicular to the direction of flow. The phenomenon is pronounced during circular Couette flow caused by the centrifugal force. The extensional strain rate induces stretching of polymer chains, resulting in their stiffening caused by storage of elastic energy. Polymer stiffening is capable of destabilising the flow, even for lower (or moderate) Reynolds numbers, by generating additional frictional effects accompanied by energy dissipation. This type of turbulence is known as elasto-inertial turbulence.
- Migrating epithelial collectives are highly complex systems capable of self-organising. The low-Reynolds turbulence in these systems represents a consequence of the viscoelasticity caused by collective cell migration and the cellular ability to adapt to micro-environmental conditions. Epithelial cells establish strong E-cadherin-mediated cell–cell adhesion contacts, which enable them to accumulate mechanical stress accompanied by the elastic energy caused by cell movement. An inhomogeneous distribution of cell mechanical stress, accompanied by epithelial surface tension, cell velocity and packing density. is a hallmark of migrating epithelial collectives. The response of cells under stress conditions includes an interplay of biological processes such as: the remodelling of cell–cell and cell–matrix adhesion contacts; contact inhibition of locomotion; the cell-jamming state transition; and the epithelial-to-mesenchymal transition. These processes can decrease undesirable stress within epithelial systems, which can supress and even stop their movement.
- In the context of low-Reynold turbulence, two model systems are considered: the fusion of two epithelial aggregates and free extension of epithelial monolayers on a substrate matrix. The driving force for the fusion of two cell aggregates is the surface tension force, while the resistive force is the viscoelastic force. Imbalance between these forces can result in long-time inertial effects (i.e. effective inertia) accompanied by oscillations of cell velocity. The cell monolayers undergo oscillatory wetting. This is caused primarily by changes in the epithelial surface tension and the accumulation of cell residual stress, which are responsible for the generation of the long-time inertial effects.

## Data Availability

Not applicable.
